# Effect of light wavelength on hot spring microbial mat biodiversity

**DOI:** 10.1371/journal.pone.0191650

**Published:** 2018-01-30

**Authors:** Akifumi Nishida, Vera Thiel, Mayuko Nakagawa, Shotaro Ayukawa, Masayuki Yamamura

**Affiliations:** 1 Department of Computational Intelligence and Systems Science, Tokyo Institute of Technology, Kanagawa, Japan; 2 Department of Biological Sciences, Tokyo Metropolitan University, Tokyo, Japan; 3 Earth-Life Science Institute, Tokyo Institute of Technology, Tokyo, Japan; 4 Waseda Research Institute for Science and Engineering, Waseda University, Tokyo, Japan; 5 Department of Computer Science, Tokyo Institute of Technology, Kanagawa, Japan; Universita degli Studi di Milano-Bicocca, ITALY

## Abstract

Hot spring associated phototrophic microbial mats are purely microbial communities, in which phototrophic bacteria function as primary producers and thus shape the community. The microbial mats at Nakabusa hot springs in Japan harbor diverse photosynthetic bacteria, mainly *Thermosynechococcus*, *Chloroflexus*, and *Roseiflexus*, which use light of different wavelength for energy conversion. The aim of this study was to investigate the effect of the phototrophs on biodiversity and community composition in hot spring microbial mats. For this, we specifically activated the different phototrophs by irradiating the mats with different wavelengths in situ. We used 625, 730, and 890 nm wavelength LEDs alone or in combination and confirmed the hypothesized increase in relative abundance of different phototrophs by 16S rRNA gene sequencing. In addition to the increase of the targeted phototrophs, we studied the effect of the different treatments on chemotrophic members. The specific activation of *Thermosynechococcus* led to increased abundance of several other bacteria, whereas wavelengths specific to *Chloroflexus* and *Roseiflexus* induced a decrease in >50% of the community members as compared to the dark conditions. This suggests that the growth of *Thermosynechococcus* at the surface layer benefits many community members, whereas less benefit is obtained from an increase in filamentous anoxygenic phototrophs *Chloroflexus* and *Roseiflexus*. The increases in relative abundance of chemotrophs under different light conditions suggest a relationship between the two groups. Aerobic chemoheterotrophs such as *Thermus* sp. and *Meiothermus sp*. are thought to benefit from aerobic conditions and organic carbon in the form of photosynthates by *Thermosynechococcus*, while the oxidation of sulfide and production of elemental sulfur by filamentous anoxygenic phototrophs benefit the sulfur-disproportionating *Caldimicrobium thiodismutans*. In this study, we used an experimental approach under controlled environmental conditions for the analysis of natural microbial communities, which proved to be a powerful tool to study interspecies relationships in the microbiome.

## Introduction

Phototrophic microbial mats are multi-layered biofilms consisting of phototrophic and chemotrophic bacteria that form in illuminated, undisturbed habitats such as hot springs, shallow sea floors, and salt lakes [1]. Hot spring microbial mats are purely microbial ecosystems owing to their elevated temperatures [2,3], and can be found all over the world. In particular, various phototrophic bacteria and coexisting chemotrophic bacteria in the mats in Nakabusa hot springs, Japan, have been studied extensively [2,4–7].

**Fig 1:**
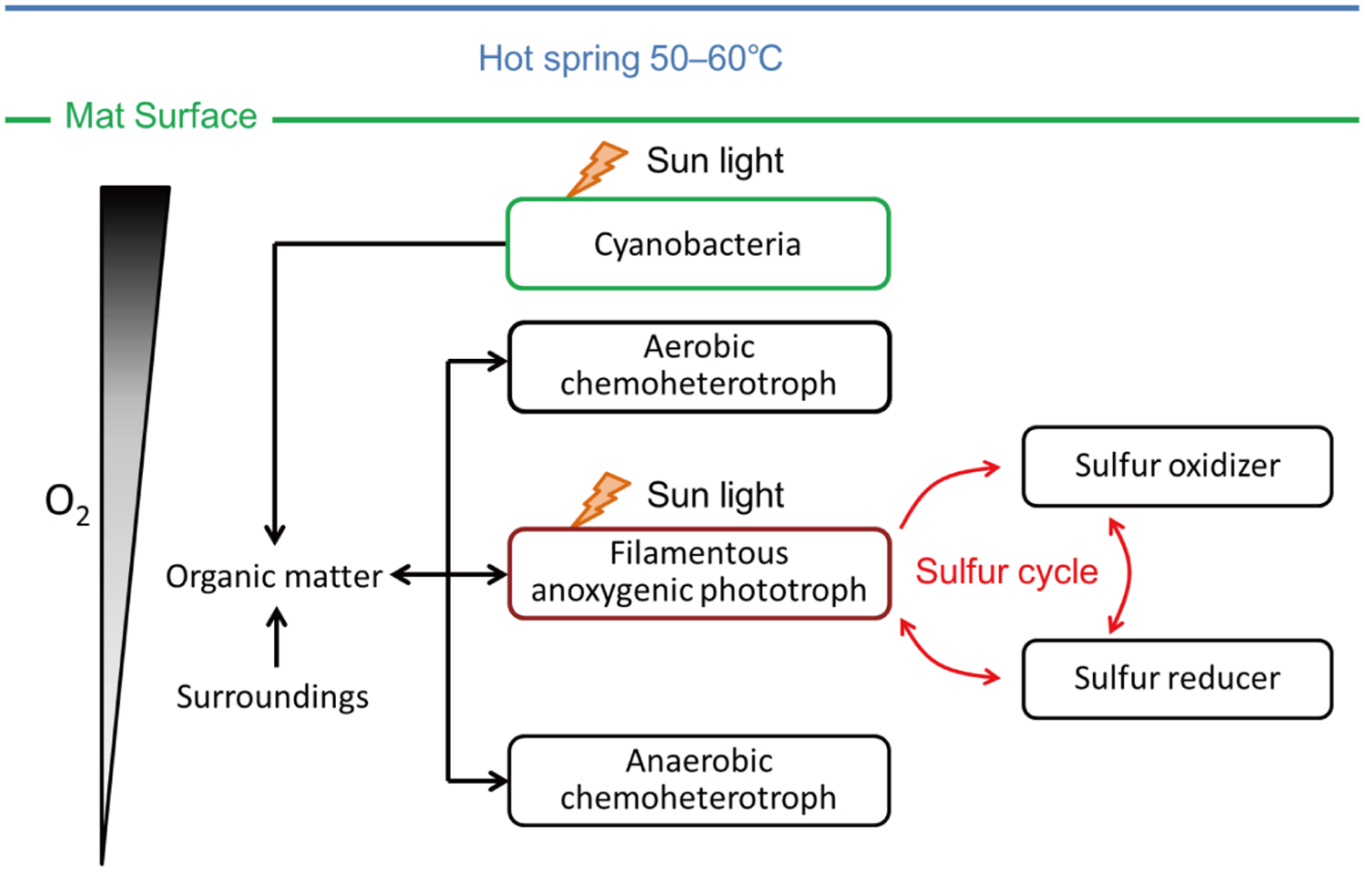
Schematic representation of a phototrophic microbial mat. Photosynthetic cyanobacteria fix CO_2_ and excrete organic matter that can be utilized by heterotrophs. Filamentous anoxygenic phototrophs oxidize sulfide to produce elemental sulfur that can be utilized by other bacteria involved in the sulfur cycle.

Photosynthetic bacteria in microbial mats shape the microbial community and influence chemotrophic bacteria in many ways, e.g., they act as primary producers, aerobic environment producers, or sulfide consumers (Fig 1). For example, autotrophic cyanobacteria produce oxygen, organic matter, and vitamins through photosynthesis and provide an environment for aerobic heterotrophs [8–11]. Some filamentous anoxygenic phototrophs such as *Chloroflexus* spp. play a crucial role in the natural sulfur cycle by oxidizing sulfide to elemental sulfur [5,7,12,13]. Transcriptomic and metabolomic studies of microbial mats confirmed the exchange of organic carbon, O_2_, and nitrogen between photoautotrophic and photoheterotrophic bacteria [14–16]. These findings support a light-dependent relationship between photosynthetic and chemotrophic bacteria. Although reports comparing the effect of environment on diel cycling or between different hot springs provide valuable information, they represent a purely observational approach. Thus, experimental studies using natural communities such as the one presented here will expand current knowledge about the environmental impact on the interspecies relationships shaping microbial mat communities.

Based on the different absorption maxima of various phototrophs, we hypothesize that irradiating microbial mats with specific light wavelengths will activate the corresponding photosynthetic bacteria to subsequently impact community composition. In the Nakabusa hot springs, oxygenic, photosynthetic cyanobacteria (genus *Thermosynechococcus*) occur in the surface layers of microbial mats at temperatures of 48–62°C [6], whereas phototrophic Chloroflexi (genera *Chloroflexus* and *Roseiflexus*) are found underneath the cyanobacterial layer [2,6,17]. These photosynthetic bacteria each utilize different light wavelengths; cyanobacteria mostly absorb light around 625 and 680 nm via phycobilin and chlorophyll (Chl) *a*, respectively [18], whereas *Chloroflexus* and *Roseiflexus* primarily absorb wavelengths of around 740 and 880 nm via bacteriochlorophyll (BChl) *c* [19,20] and BChl *a*, respectively [21,22]. Cyanobacterial photosynthesis provides organic matter and oxygen to the surrounding microenvironment [8–11]. *Chloroflexus* spp. are reported to grow photoautotrophically via the 3-hydroxypropionate pathway and subsequently circulate organic matter to adjacent heterotrophic bacteria [19,23–25]. Although *Roseiflexus castenholzii* has not been shown to grow photoautotrophically, its ability to fix inorganic carbon during autotrophic or mixotrophic growth is assumed, given that it harbors the complete gene set required for the 3-hydroxypropionate pathway [26,27]. Furthermore, *Roseiflexus* sp. RS-1 isolated from hot springs in Yellowstone National Park (YNP) was demonstrated to grow photoautotrophically or photomixotrophically in situ by stable carbon isotope and metatranscriptome analysis [14,26,28]. Based on this evidence, we hypothesize that irradiating microbial mats with defined light wavelengths utilized by *Thermosynechococcus*, *Chloroflexus*, and *Roseiflexus* spp. will specifically enrich the corresponding phototrophs, as well as their commensal chemotrophs. In the present study, natural microbial mat communities were incubated under controlled light conditions in situ and analyzed by 16S rRNA gene sequencing, which serves as a powerful approach to study interspecies relationships in microbiomes.

## Materials and methods

### Sample site and sample collection

Nakabusa hot spring is located in the Northern Japanese Alps (Nagano, Japan) at an elevation of approx. 1,400 m above mean sea level. It is one of the best investigated hot springs in the world and contains several outlets. High source temperatures of up to 90°C, high sulfide levels and subsequent temperature gradients in the outflow channels lead to habitats for multiple types of communities; e.g., chemotrophic streamers, as well as purely anoxygenic, and oxygenic phototrophic microbial mats. Nakabusa hot spring contains two characteristic sites, one in which the water leaves the ground at an elevated location and flows down a concrete wall, where colorful microbial communities develop ('Wall site'; 36°23’20”N, 137°44’53”E; Panel A in S1 Fig). At a second site approximately 300 m away from the Wall site, hot spring water surfaces at ground level and forms small pools and outflow channels over sandy ground ('Site B'; 36°23’33”N, 137°44’53”E; Panel B in S1 Fig). The chemical and gas compositions in an outlet at the Wall site show that the concentrations of nitrite, nitrate, and O_2_ are below the detection limit (<0.01 mg/L for nitrogen compounds) [29]. Sulfide concentrations at the Site B is ~120 μM, similar to that of the Wall site [30]. The hot spring is slightly alkaline and sulfur compound-rich; therefore, the microbial community related to sulfur cycle has been particularly investigated [5,7,31]. The mats at temperatures ≤63°C are dominated by cyanobacteria and anoxygenic phototrophic Chloroflexi [5,7], and similar to mats observed in other Japanese and YNP alkaline hot springs [2,32].

We sampled approximately 40 cm^3^ (10 × 4 × 1 cm^3^) of a microbial mat at the Wall site on May 30th, 2016 (S2 Fig). The temperature and pH at the sampling spot were 56°C and 8.7, measured using a FUSO-370 RTD Thermometer (Fuso, Tokyo, Japan) and PH-6600 (Custom Corporation, Tokyo, Japan), respectively. The sample was manually homogenized by vigorous shaking in a 50-mL tube and then dispensed into ~1.6 cm^3^ (2 × 1.6 × 0.5 cm^3^) cavities of the triplicate light-irradiating devices covered with a clear acrylic board (S3 Fig). The remainder of the bacterial suspension, accounting for ~15 cm^3^ of the original mat, was transferred to 2-mL reaction tubes (Eppendorf, Hamburg, Germany) for DNA isolation and 16S rRNA gene analysis. The devices were then placed in a newly dug horizontal hot spring channel at the Site B; mounting the experimental set up at the Wall site was not possible due to its vertical location (S1 Fig). The average temperature and pH at the spot in which the devices were placed were 56–50°C and 7.3, respectively (S4 Fig). After irradiation under experimental conditions for 20 days, the mats were collected using autoclaved tweezers and mixed with biofilms that developed on the surface of the clear cover. Hot spring water (1 L) was collected from the incubation site surrounding device 1 on days 0, 7, 14, and 20 for 16S rRNA gene sequencing. Due to limitation of experimental mat volume, mat samples were obtained only at the end of the experiment and temporal observations could not be achieved. All measurements were performed in triplicate (#1–3). Due to the serial positioning of the triplicates in the hot spring stream, the microbial communities experienced slightly different ambient temperatures (55°C, 53°C, 51°C for device 1–3, respectively; S4 Fig).

### Light irradiation

Microbial mats were irradiated with light at specific wavelengths using a device developed by our group consisting of a black acrylic board (Shinkolite, Mitsubishi Rayon Co., Ltd., Tokyo, Japan) with five tracks for dark, 625 nm, 730 nm, 890 nm, and all three wavelengths combined (S5 Fig). Homogenized microbial mat samples were placed in the cavities and covered with a clear acrylic board. The mats were continuously irradiated for 20 days using LEDs specific for each wavelength (5 mA; OSR5CA5B61P for 625 nm, SX534IR-730 for 730 nm, and TSHF5410 for 890 nm; all from Akizuki Denshi Tsusho, Co Ltd., Tokyo, Japan). The incubation period of 20 days was chosen based on the doubling time of the three phototrophs, which is approximately 24 h [23,28,33]. Furthermore, partial mat recovery was observed in the initial mat sampling spot over that time as shown in S6 Fig. The time frame was thus expected to be sufficient for the observation of specific differences between the conditions. The distance between the LEDs and microbial mat surface was 20 mm. Due to a size limitation of the spectroradiometer (OL-750, Gooch & Housego, Ilminster, UK), light intensity at 20 mm distance was calculated by inverse-square law using intensity values measured at 30 and 50 cm distance (S7 Fig). The light intensity for the 625, 730, and 890 nm LEDs at 20 mm was approximately 0.2, 0.4, and 0.1 W/m^2^/nm, respectively. Due to a ±30 nm irradiation range for each LED, 625 nm instead of 680 nm was chosen to specifically activate photosynthesis in *Thermosynechococcus* spp. to avoid overlap with the in-situ absorbance of BChl *c* (S7 Fig).

### DNA isolation, PCR amplification, and sequencing

Genomic DNA was isolated from the microbial mat and hot spring water samples using the PowerBiofilm DNA and PowerWater Sterivex DNA isolation kits (Mo Bio Laboratories, Carlsbad, CA, USA), respectively. An area spanning the V3 and V4 variable regions of the 16S rRNA gene was amplified using KOD FX Neo polymerase (Toyobo, Osaka, Japan) according to the manufacturer’s protocol (primer information is in S1 Table). PCR products were cleaned using the Wizard SV Gel and PCR Clean-Up System (Promega, Madison, WI, USA). The cleaned samples were then loaded onto a MiSeq reagent cartridge for paired-end sequencing and automated clustering with MiSeq (Illumina, San Diego, CA, USA) with dual index reads and a 300-bp read length at Earth-Life Science Institute of Tokyo Institute of Technology.

### Taxonomic classification based on 16S rRNA gene sequences

The paired-end reads of the partial 16S rRNA gene sequences were clustered by 97% nucleotide identity, and then assigned taxonomic information using the SILVA database [34]. The steps for data processing and assignment were as follows: (i) trimming sequences with a quality score from the 3'-end with a threshold score of 20 in PRINSEQ [35]; (ii) removing reads of the PhiX genome with Bowtie2 [36]; (iii) trimming primer sequences at a 20% error tolerance in cutadapt [37]; (iv) joining paired-end reads with QIIME [38]; (v) filtering reads with a quality score by usearch [39] with total expected errors set at 1; (vi) dereplicating reads with 100% identity by usearch; (vii) removing singletons and chimeras by usearch; (viii) clustering operational taxonomic units (OTUs) with 97% identity; and (ix) assigning taxonomic information to each OTU using uclust with SILVA taxonomy data (SILVA 123 QIIME compatible database, taxonomy 7 levels, last modification May 2016) for 97% identity in QIIME. We used all processed sequences for clustering of OTUs and relative abundance of OTU sequences in each sample without subsampling process. The numbers of row and processed sequences are shown in S2 Table and *p*-values were calculated by two-tailed paired *t*-test for comparison among experimental mats and two-tailed Welch’s *t*-test for comparison among experimental mat, initial mat, and hot spring water.

The change in relative abundance was analyzed for each OTU observed. Only the selection of OTUs will be discussed here. We evaluated those members that show clear or notably high response to the experimental conditions, and, based on the assumption that more abundant mat members will have a higher ecological significance, specifically analyzed more abundant (≥1% averaged relative abundance in any light condition) mat community members. For the calculation of the average, we summed up relative abundance of OTU in triplicate, and then, divide the summation by three. The 1% cutoff was chosen somewhat arbitrarily based on experiences of previous studies [40] and based on the average relative abundance of their representing amplicon sequences. Further OTU sequences representing possible phototrophic bacteria and/or those showing considerable changes in relative abundance (see the section "Effect of light on specific microbial mat members" for the criterion) were included in an in-depth analysis to prevent the bias introduced by focusing on abundance only. The sequences were taxonomically identified by comparison to known sequences in NCBI nr/nt databases by BLAST search [41] and phylogenetic analysis using the ARB software package [42]. Imported sequences were aligned automatically using the pt_server database and manually corrected based on secondary structure information. Initial phylogenetic affiliations were obtained by adding the aligned sequences to the tree_SSURefNR99_1200_slv_123 tree backbone implemented in SILVA (SSU Ref. NR 123, released July 2015). Phylogenetic trees were generated based on the maximum likelihood method using the phyML software included in the ARB package. The inferred confidence was based on 100 bootstrap replicates, and only values of >50 were shown in phylogenetic trees. Only sequences with length ≥1,000 nt were used for phylogenetic calculations. Short amplicon sequences (<1000 nt) from the present or previous studies, as well as partial sequences of uncultivated relatives, were added to trees using the ARB parsimony method without changing the tree topology.

### Biodiversity analysis

Bacterial biodiversity was assessed by Shannon Diversity Index, Chao1, observed OTUs, and equitability based on 97% nucleotide sequence identity. These values and rarefaction curves were calculated by QIIME [38] with a depth of 90,000 and a trial of 10. *P*-values were calculated by two-tailed paired *t*-test for comparison among experimental mats and two-tailed Welch’s *t*-test for comparison among experimental mat, initial mat, and hot spring water. Furthermore, wavelength-induced differences in bacterial community composition were determined by calculating the relative abundance for each OTU under different light conditions with respect to controls grown in the dark using the following equation: *F*_*i*,*j*,*k*_ = *R*_*i*,*j*,*k*_*/R*_*i*,*0*,*k*_, where *F*_*i*,*j*,*k*_ indicates the fold change in the relative abundance of samples grown in light (*R*_*i*,*j*,*k*_) and dark (*R*_*i*,*0*,*k*_) conditions, and *i*, *j*, and *k* represent the OTU ID, light condition (0: dark, 1: 625 nm, 2: 730 nm, 3: 890 nm, 4: combined light), and device ID (1–3), respectively. The fold-change analysis was restricted to OTUs with ≥10 reads, as smaller values would result in less reliable data with regard to relative changes in species abundance.

## Results and discussion

### Observed differences after experimental cultivation in situ

**Fig 2:**
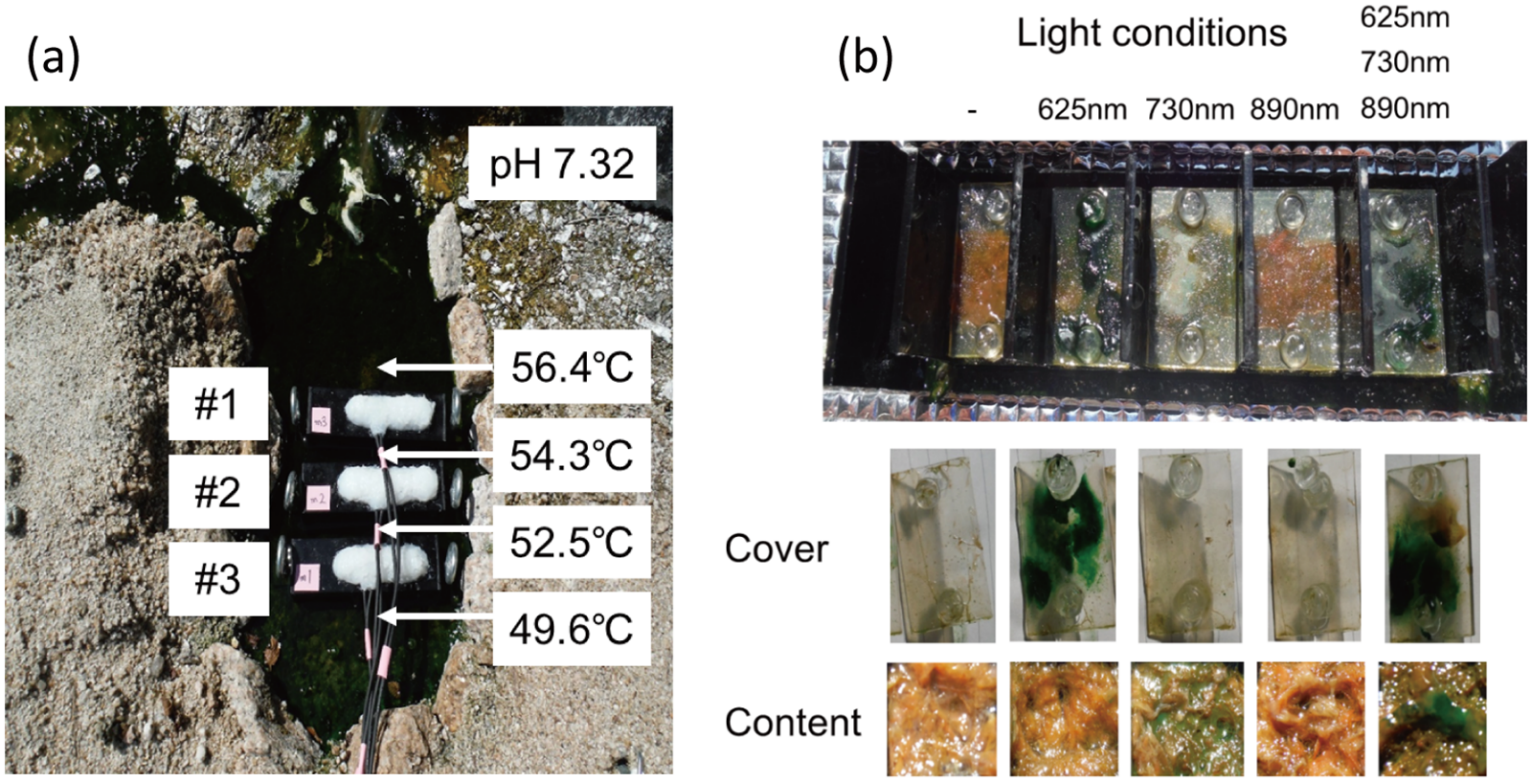
Microbial mat cultivation with specific light wavelengths. (a) Average temperature and pH during mat cultivation. (b) Images of microbial mats cultivated at different wavelengths and controls.

**Fig 3:**
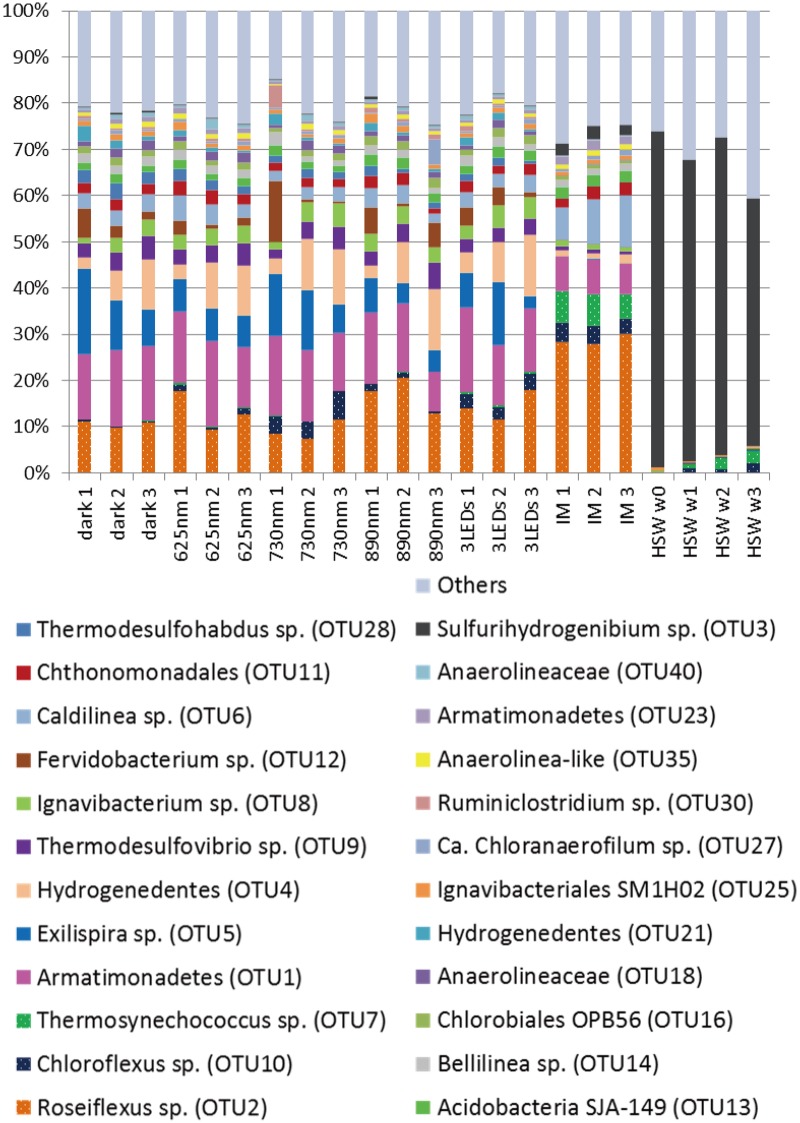
Differences in relative abundance of community members in microbial mats and hot spring water. The relative abundance of community members was examined in microbial mats before (indicated as "IM") and after irradiation in triplicates with light at 625, 730, or 890 nm for 20 days. Samples cultivated in the dark and with combined light served as controls. Hot spring water around the devices was also sampled on days, 0, 7, 14, and 20 (indicated as "HSW" with w0, w1, w2, and w3, respectively). Averaged abundance in triplicates of ≥1% in at least one experimental condition, the three phototrophs, and *Sulfurihydrogenibium* sp. (OTU3) dominant in hot spring water are shown.

**Fig 4:**
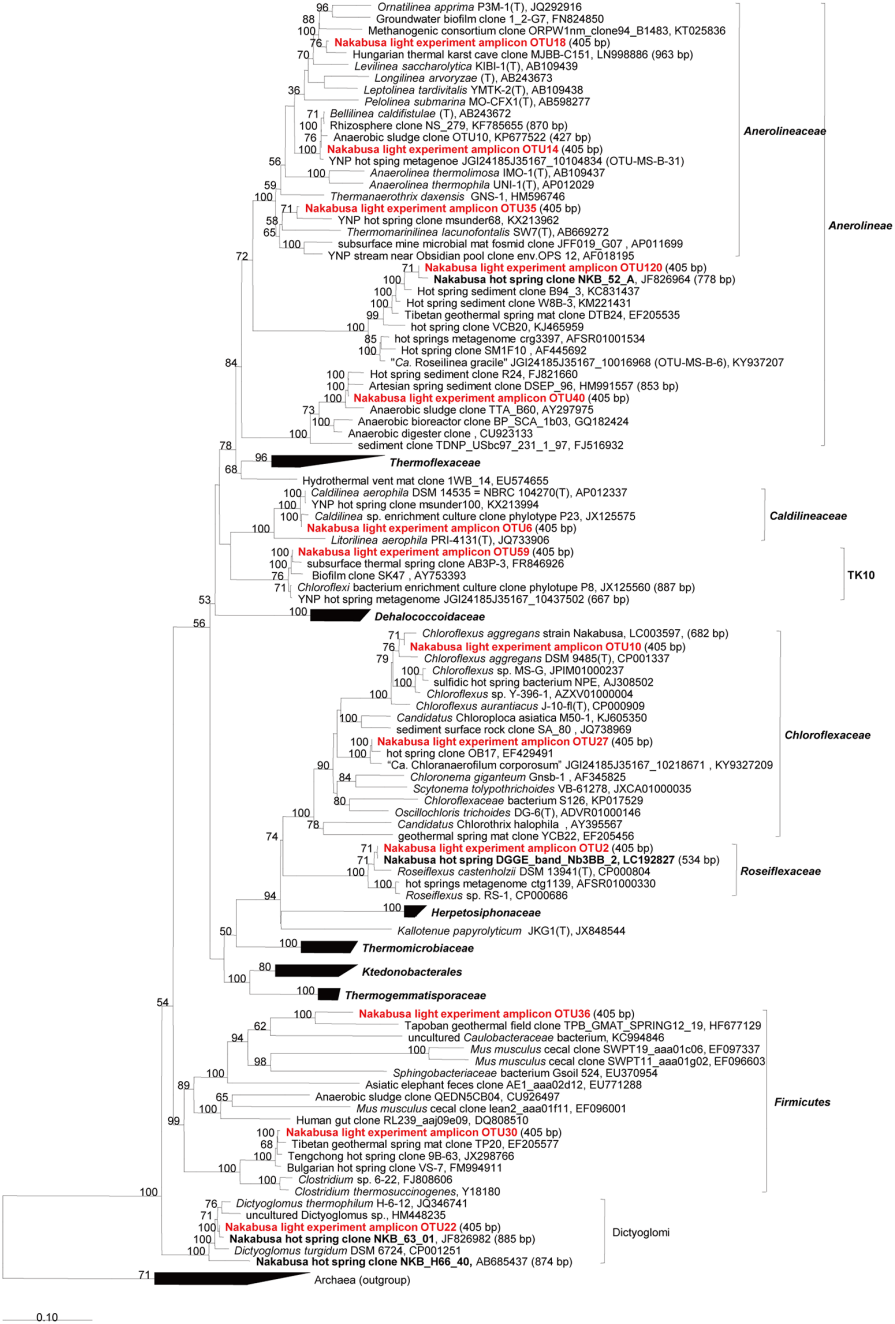
Phylogenetic tree based on abundant sequences in initial/experimental mats and increased/decreased sequences associated with specific light wavelengths for the phylum Chloroflexi, Firmicutes, and Dictyoglomi. The tree shows sequences obtained from the Nakabusa microbial mats in previous studies (bold) and this study (bold, red).

**Fig 5:**
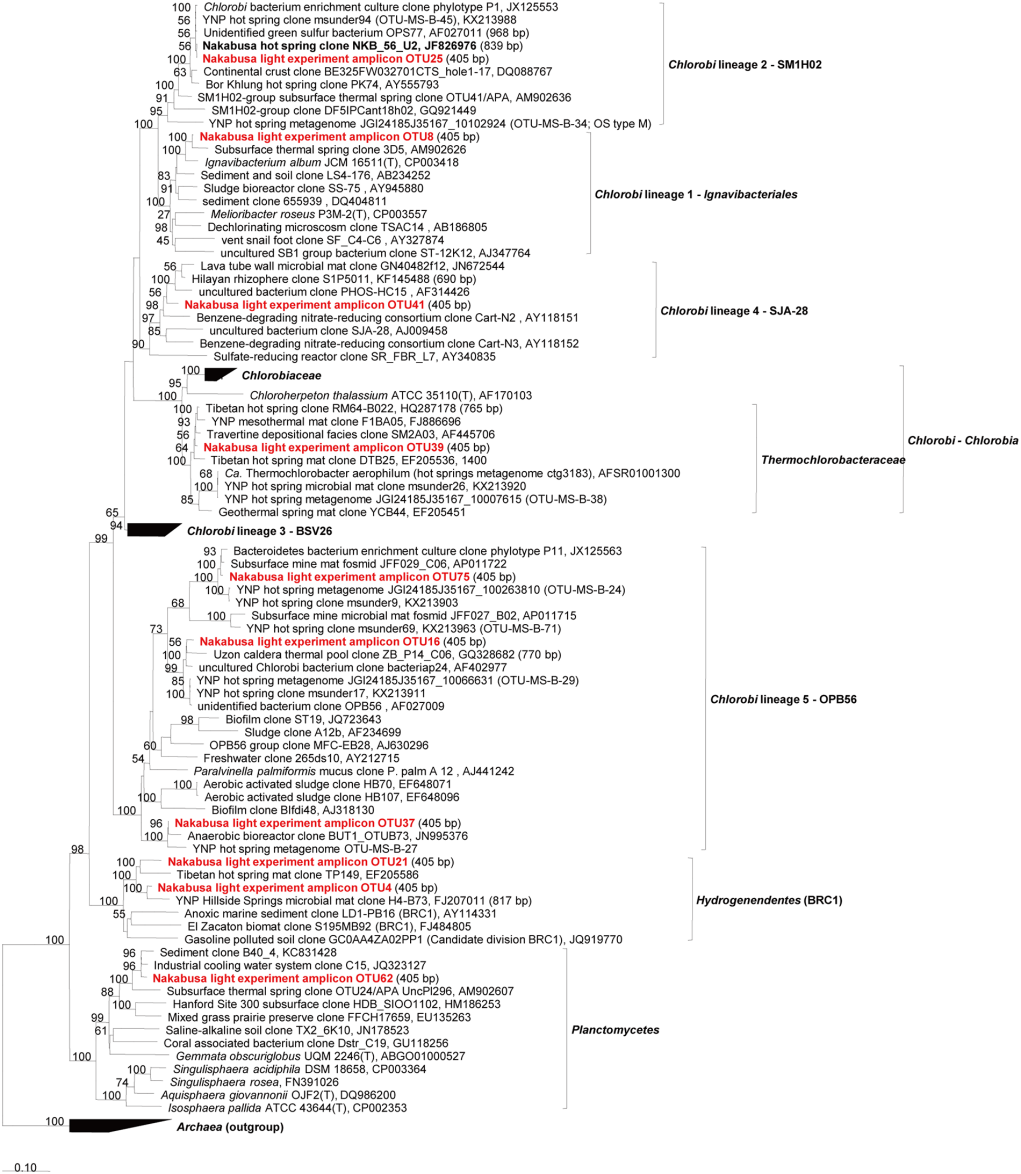
Phylogenetic tree based on abundant sequences in initial/experimental mats and increased/decreased sequences associated with specific light wavelengths for the phylum Chlorobi, Hydrogenedentes and Planctomycetes. The tree shows sequences obtained from the Nakabusa microbial mats in previous studies (bold) and this study (bold, red).

**Fig 6:**
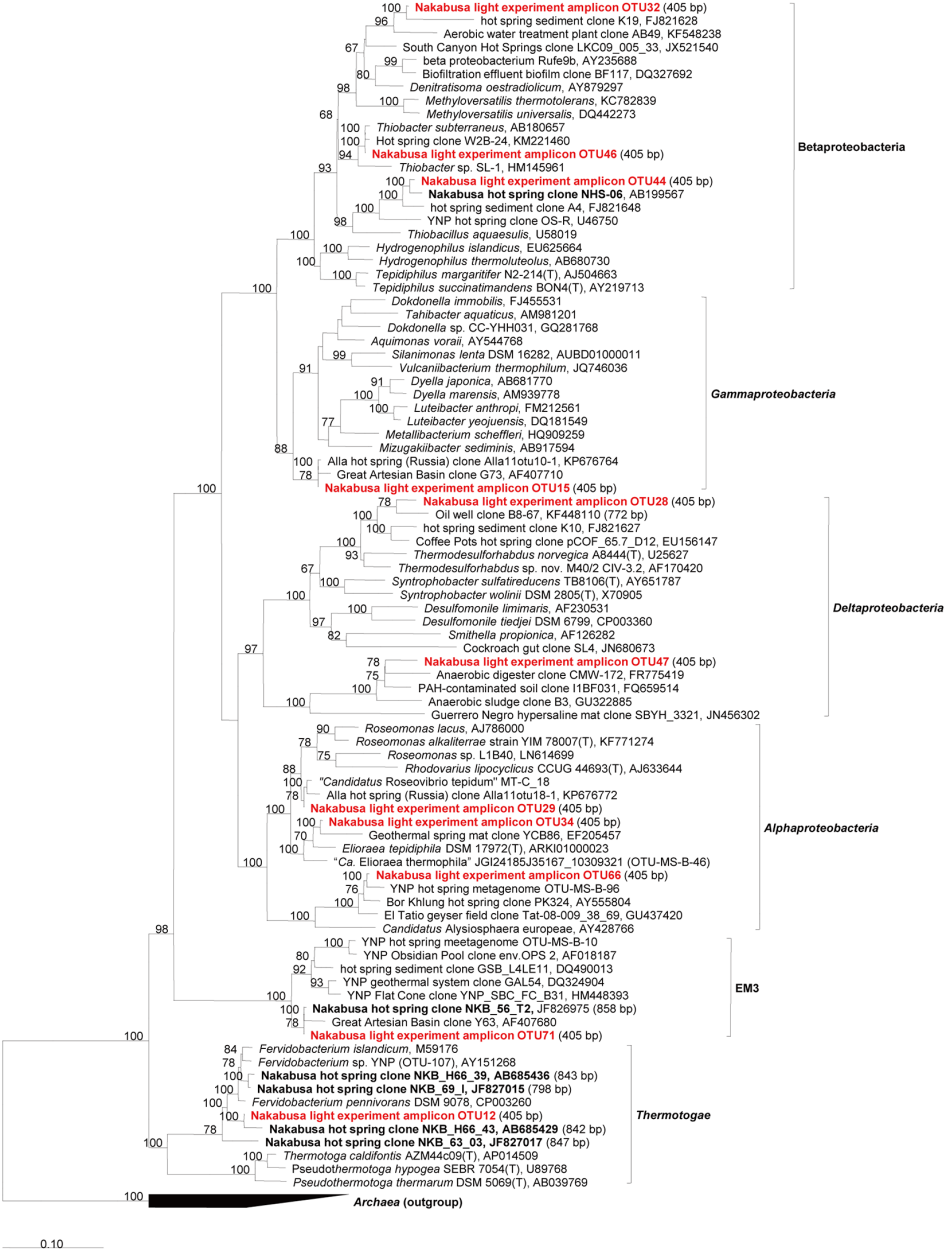
Phylogenetic tree based on abundant sequences in initial/experimental mats and increased/decreased sequences associated with specific light wavelengths for the phylum Proteobacteria, Thermotogae and EM3. The tree shows sequences obtained from the Nakabusa microbial mats in previous studies (bold) and this study (bold, red).

**Fig 7:**
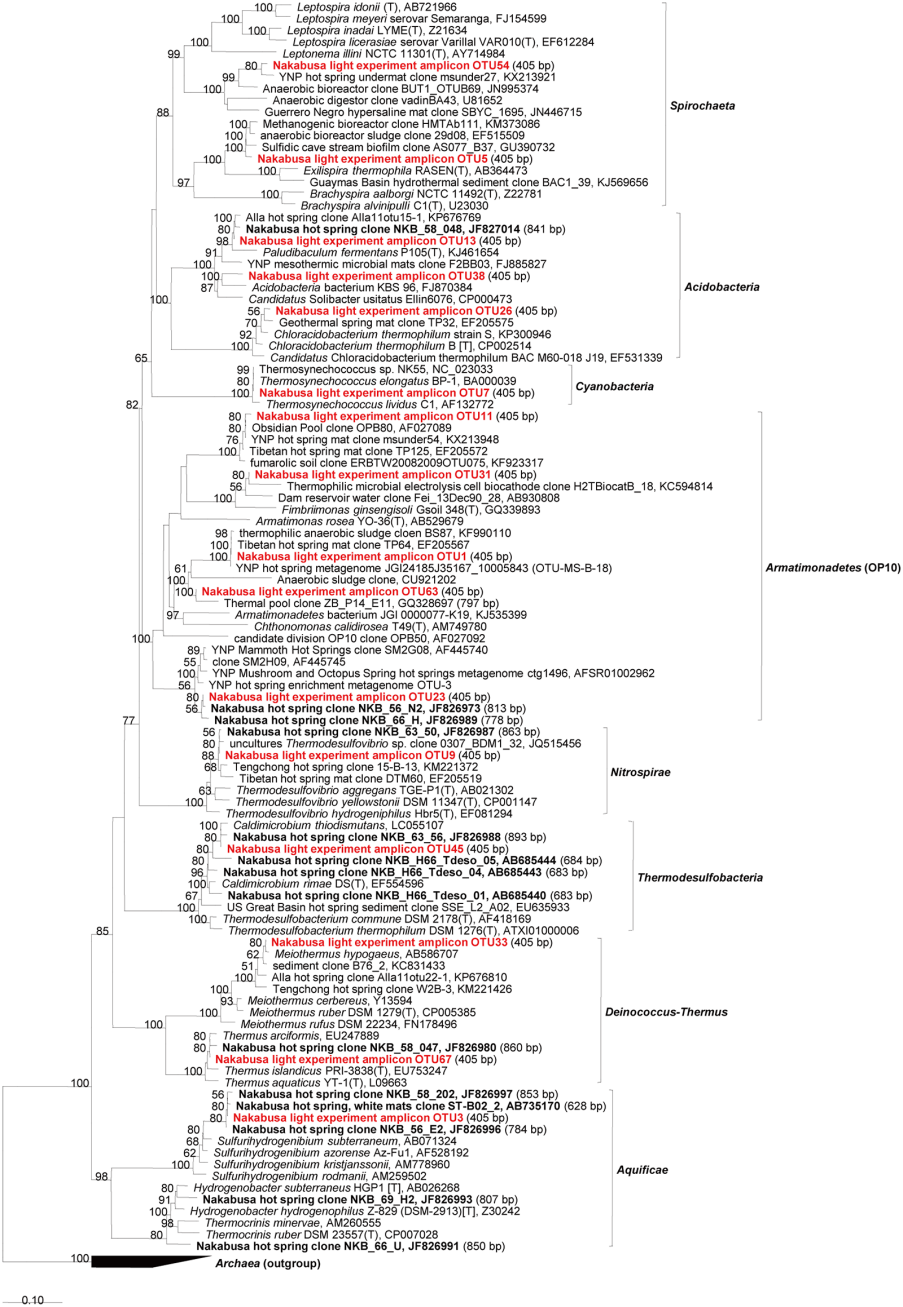
Phylogenetic tree based on abundant sequences in initial/experimental mats and increased/decreased sequences associated with specific light wavelengths for various other phyla. The tree shows sequences obtained from the Nakabusa microbial mats in previous studies (bold) and this study (bold, red).

In this study, we used a controlled approach with defined light wavelengths to examine the effect of the physiological activity of different phototrophic bacterial members on diversity and community composition in phototrophic microbial mats. Hot spring-associated phototrophic microbial mat communities were sampled, homogenized and incubated in-situ under varying light conditions to specifically stimulate three different phototrophic members, i.e., *Thermosynechococcus*, *Chloroflexus* and *Roseiflexus*, of the mat community. Three different wavelengths (625 nm, 730 nm, and 890 nm) were used to specifically activate one of the phototrophs under each condition. Dark and combined light conditions served as control treatments. The mats were incubated in-situ in natural hot spring water under controlled, constant LED light conditions (Fig 2). After 20 days of incubation, the microbial mats were sampled and the microbial community was analyzed using 16S rRNA gene amplicon sequencing analysis (S1 and S2 Datasets). Abundant members in experimental mats (averaged relative abundance ≥1%), the three phototrophs, and *Sulfurihydrogenibium* sp. (OTU3, 99% nt identity) dominant in hot spring water are shown in Fig 3 and Tables 1 and 2. Furthermore, they were also subjected to identification via BLAST and phylogenetic analysis (Figs 4–7). Although OTU sequences related to three phototrophs, i.e., *Thermosynechococcus* sp., *Chloroflexus aggregans*, and *Roseiflexus castenholzii*, increased in corresponding light conditions, we will discuss them in more detail in the section "Effect of light wavelength on phototrophic bacteria" below. At first, we discuss the visual differences of the experimental mats with the 16S rRNA gene amplicon sequencing analysis.

**Table 1:** Abundant members with relative abundance ≥1% in the experimental mats, targeted cyanobacteria, and dominant bacterium in hot spring water. Nearest neighbors of the sequence list based on BLAST search from all NCBI database sequences and type material.

OTU ID	Taxa	Relative abundance (average)						BLAST (all)	Acc. No.	Identity	e-value
		Dark	625nm	730nm	890nm	3LEDs	IM^†^	BLAST (type strain)			
OTU2	Chloroflexi, Roseiflexus	10.8%	13.4%	9.3%	17.3%	14.7%	28.9%	Roseiflexus castenholzii strain DSM 13941	CP000804.1	100%	0
								Roseiflexus castenholzii strain DSM 13941	CP000804.1	100%	0
OTU10	Chloroflexi, Chloroflexus	0.3%	1.1%	4.6%	1.0%	3.1%	3.8%	hot spring uncultured bacterium clone NKB_H66_01	AB685439.1	100%	0
								Chloroflexus aggregans DSM 9485	CP001337.1	99%	0
OTU7	Cyanobacteria, Thermosynechococcus	0.0%	0.2%	0.1%	0.1%	0.3%	6.3%	Thermosynechococcus sp. NK55	CP006735.1	100%	0
								Coleofasciculus chthonoplastes strain SAG 2209	NR_125521.1	91%	2E-150
OTU1	Armatimonadetes, unc.	15.6%	15.7%	15.0%	12.9%	15.0%	7.2%	hot spring uncultured bacterium clone TP54	EF205567.2	100%	0
								Pelotomaculum thermopropionicum SI	AP009389.1	85%	4E-113
OTU5	Spirochaetae, Exilispira	12.5%	7.1%	10.9%	5.5%	8.0%	0.0%	thermophilic uncultured bacterium clone HMTAb111	KM373086.1	99%	0
								Exilispira thermophila strain RASEN	NR_041644.1	85%	5E-112
OTU4	Hydrogenedentes, unc.	6.5%	7.9%	8.7%	8.2%	8.8%	1.5%	hot spring ucultured bacterium clone H4-B73	FJ207011.1	99%	0
								Aliifodinibius sediminis strain YIM J21	NR_118429.1	80%	3E-69
OTU9	Nitrospirae, Thermodesulfovibrio	4.0%	4.0%	3.6%	4.2%	3.1%	0.7%	hot spring uncultured bacterium clone NKB_63_50	JF826987.1	100%	0
								Thermodesulfovibrio yellowstonii DSM 11347	CP001147.1	97%	0
OTU8	Chlorobi, Ignavibacterium	2.6%	3.4%	3.6%	3.6%	4.1%	1.2%	hot spring uncultured bacterium clone OTU42/APA	AM902626.1	98%	0
								Ignavibacterium album JCM 16511	CP003418.1	97%	0
OTU12	Thermotogae, Fervidobacterium	3.6%	1.9%	4.8%	3.9%	3.0%	0.0%	hot spring uncultured bacterium clone NKB_H66_43	AB685429.1	100%	0
								Fervidobacterium riparium strain 1445t	NR_108234.1	99%	0
OTU6	Chloroflexi, Caldilinea	3.4%	4.3%	2.7%	3.4%	3.4%	9.4%	Caldilinea tarbellica strain D1-25-10-4	NR_117797.1	100%	0
								Caldilinea tarbellica strain D1-25-10-4	NR_117797.1	100%	0
OTU11	Atmatimonadetes, Chthonomonadales	2.3%	2.7%	1.7%	2.1%	2.1%	2.6%	hot spring uncultured bacterium clone TP125	EF205572.1	99%	0
								Thermanaerovibrio acidaminovorans strain DSM 6589	NR_074520.1	85%	3E-109
OTU28	Deltaproteobacteria, Thermodesulforhabdus	3.0%	2.2%	1.7%	1.5%	1.4%	0.0%	thermophilic unc. delta proteobacterium clone B8-67	KF448110.1	100%	0
								Thermodesulforhabdus norvegica strain A8444	NR_025970.1	92%	5E-157
OTU13	Acidobacteria, SJA-149	1.7%	1.7%	1.6%	2.2%	2.1%	2.4%	hot spring uncultured bacterium clone Alla11otu15-1	KP676769.1	100%	0
								Paludibaculum fermentans strain P105	NR_134120.1	95%	2E-180
OTU14	Chloroflexi, Bellilinea	1.8%	1.9%	1.7%	1.7%	1.9%	1.5%	thermophilic uncultured bacterium clone OTU10	KP677522.1	100%	0
								Bellilinea caldifistulae strain GOMI-1	NR_041354.1	100%	0
OTU16	Chlorobi, OPB56	1.6%	1.4%	1.3%	2.0%	1.7%	0.9%	hot spring uncultured bacterium clone ZB_P14_C06	GQ328682.1	99%	0
								Thermosulfidibacter takaii ABI70S6	AP013035.1	82%	5E-87
OTU18	Chloroflexi, Anaerolineaceae	1.8%	1.8%	1.5%	1.2%	1.2%	0.0%	thermophilic uncultured bacterium clone MJBB-C151	LN998886.1	99%	0
								Bellilinea caldifistulae strain GOMI-1	NR_041354.1	94%	2E-170
OTU21	Hydrogenedentes, unc.	2.0%	1.0%	1.2%	1.2%	1.2%	0.0%	hot spring uncultured bacterium clone TP149	EF205586.1	93%	2E-167
								Paracoccus laeviglucosivorans strain 43P	NR_145640.1	83%	3E-64
OTU25	Chlorobi, SM1H02	1.1%	1.2%	0.9%	1.3%	1.1%	0.9%	hot spring uncultured bacterium clone NKB_56_U2	JF826976.1	100%	0
								Ignavibacterium album strain JCM 16511	NR_074698.1	88%	3E-134
OTU27	Chloroflexi, "Ca. Chloranaerofilum"	0.6%	0.7%	0.6%	2.2%	0.7%	0.9%	hot spring uncultured Chloroflexi bacterium clone OB17	EF429491.2	100%	0
								Oscillochloris trichoides strain DG-6	NR_114470.1	92%	2E-160
OTU30	Firmicutes, Ruminiclostridium	0.6%	0.4%	1.8%	0.9%	0.6%	0.1%	thermophilic uncultured bacterium clone 9B-63	JX298766.1	99%	0
								Ruminiclostridium thermocellum strain ATCC 27405	NR_074629.1	93%	1E-168
OTU35	Chloroflexi, Anaerolineaceae	0.9%	1.0%	0.8%	0.8%	0.7%	1.1%	hot spring uncultured bacterium clone msunder68	KX213962.1	99%	0
								Thermomarinilinea lacunifontana strain SW7	NR_132293.1	92%	5E-162
OTU23	Armatimonadetes, unc.	0.9%	1.1%	0.7%	0.7%	0.8%	1.8%	hot spring uncultured bacterium clone NKB_56_N2	JF826973.1	100%	0
								Thermosediminibacter oceani DSM 16646	CP002131.1	85%	5E-112
OTU40	Chloroflexi, Anaerolineaceae	0.8%	1.2%	0.8%	0.5%	0.6%	0.3%	thermophilic unc. Chloroflexi bacterium clone DSEP_96	HM991557.1	100%	0
								Thermanaerothrix daxensis strain GNS-1	NR_117865.1	87%	2E-125
OTU3	Aquificae, Sulfurihydrogenibium	0.3%	0.2%	0.2%	0.3%	0.3%	2.5%	hot spring uncultured Aquificaceae bacterium clone ST-B02_2	AB735170.1	100%	0
								Sulfurihydrogenibium azorense strain Az-Fu1	NR_102858.1	99%	0

^†^IM: initial mat

**Table 2:** Standard deviations and coefficient of variations between triplicates for Fig 3 listing abundant members in microbial mats.

OTU ID	Taxa	Standard deviation						Coefficient of variation					
		Dark	625nm	730nm	890nm	3LEDs	IM^†^	Dark	625nm	730nm	890nm	3LEDs	IM^†^
OTU2	Chloroflexi, Roseiflexus	0.7%	4.2%	2.2%	3.9%	3.2%	1.1%	0.06	0.32	0.24	0.23	0.22	0.04
OTU10	Chloroflexi, Chloroflexus	0.1%	0.5%	1.3%	0.6%	0.4%	0.4%	0.41	0.44	0.29	0.57	0.14	0.1
OTU7	Cyanobacteria, Thermosynechococcus	0.0%	0.1%	0.0%	0.0%	0.1%	0.9%	0.67	0.23	0.76	0.11	0.22	0.15
OTU1	Armatimonadetes, unc.	1.2%	2.8%	2.4%	3.8%	2.8%	0.5%	0.08	0.18	0.16	0.29	0.19	0.07
OTU5	Spirochaetae, Exilispira	5.5%	0.1%	4.0%	1.7%	5.5%	0.0%	0.44	0.01	0.37	0.31	0.69	0.59
OTU4	Hydrogenedentes, unc.	4.2%	4.2%	4.8%	5.2%	4.4%	0.3%	0.64	0.54	0.54	0.64	0.51	0.2
OTU9	Nitrospirae, Thermodesulfovibrio	1.1%	0.6%	1.4%	1.3%	0.4%	0.0%	0.26	0.16	0.38	0.31	0.14	0.05
OTU8	Chlorobi, Ignavibacterium	1.3%	0.4%	1.8%	0.2%	1.0%	0.2%	0.49	0.11	0.5	0.06	0.24	0.15
OTU12	Thermotogae, Fervidobacterium	2.4%	1.2%	7.3%	2.8%	1.7%	0.0%	0.66	0.66	1.52	0.72	0.56	0.48
OTU6	Chloroflexi, Caldilinea	0.2%	1.2%	0.4%	1.3%	0.5%	2.1%	0.04	0.28	0.16	0.38	0.15	0.23
OTU11	Atmatimonadetes, Chthonomonadales	0.1%	0.5%	0.2%	0.8%	0.4%	0.4%	0.05	0.2	0.1	0.39	0.18	0.17
OTU28	Deltaproteobacteria, Thermodesulforhabdus	0.5%	0.4%	0.3%	0.8%	0.6%	0.0%	0.16	0.19	0.19	0.53	0.43	0.36
OTU13	Acidobacteria, SJA-149	0.2%	0.2%	0.4%	0.3%	0.3%	0.1%	0.11	0.14	0.25	0.11	0.14	0.03
OTU14	Chloroflexi, Bellilinea	0.1%	0.3%	0.9%	0.5%	0.5%	0.1%	0.06	0.15	0.54	0.27	0.24	0.04
OTU16	Chlorobi, OPB56	0.2%	0.4%	0.2%	0.1%	0.3%	0.1%	0.14	0.3	0.19	0.06	0.18	0.09
OTU18	Chloroflexi, Anaerolineaceae	0.6%	0.6%	0.8%	0.2%	0.5%	0.0%	0.36	0.33	0.53	0.16	0.39	0.46
OTU21	Hydrogenedentes, unc.	1.2%	0.4%	1.0%	0.6%	0.7%	0.0%	0.61	0.41	0.85	0.56	0.56	0.45
OTU25	Chlorobi, SM1H02	0.2%	0.4%	0.2%	0.5%	0.1%	0.1%	0.14	0.38	0.18	0.41	0.13	0.11
OTU27	Chloroflexi, "Ca. Chloranaerofilum"	0.2%	0.2%	0.2%	2.9%	0.4%	0.4%	0.33	0.35	0.26	1.35	0.54	0.39
OTU30	Firmicutes, Ruminiclostridium	0.1%	0.3%	2.6%	0.3%	0.3%	0.0%	0.17	0.86	1.43	0.32	0.59	0.22
OTU35	Chloroflexi, Anaerolineaceae	0.3%	0.1%	0.4%	0.2%	0.0%	0.1%	0.3	0.06	0.56	0.23	0.06	0.1
OTU23	Armatimonadetes, unc.	0.2%	0.1%	0.1%	0.2%	0.1%	0.2%	0.24	0.09	0.13	0.26	0.16	0.12
OTU40	Chloroflexi, Anaerolineaceae	0.3%	0.9%	0.4%	0.0%	0.1%	0.0%	0.32	0.74	0.54	0.05	0.13	0.15
OTU3	Aquificae, Sulfurihydrogenibium	0.1%	0.0%	0.1%	0.2%	0.1%	0.2%	0.42	0.14	0.39	0.59	0.33	0.09

^†^IM: initial mat

Visual differences in color were observed in microbial mats after 20 days of cultivation; their development under the different conditions is shown in Fig 2. Mats cultivated with 625-nm light harbored a thin green layer of *Thermosynechococcus* sp., as supported by 16S rRNA gene sequencing (Table 1). This layer had a thickness of <1 mm, similar to the newly formed green mats observed on the sediment surrounding the light-irradiating devices exposed to natural sunlight. The microbial mats cultivated with 730-nm light showed a ~3-mm-thick brown upper layer most likely dominated by *Chloroflexus* sp., which overlaid a ~2-mm-thick layer of orange-pink *Roseiflexus* (S8 Fig). This distribution is identical to hypersaline mats in which a *Chloroflexus* layer forms immediately on top of a concentrated layer of *Roseiflexus* as determined by FISH analysis [43]. No color differences were observed between mats cultivated with 890-nm light or in the dark; both were orange-pink, a color associated with *Roseiflexus* dominated communities [2,6]. This is not unexpected given that *Roseiflexus* can grow both photomixo/heterotrophically and chemoheterotrophically and given the observed abundance of *Roseiflexus castenholzii* OTU2 under both conditions (17%±4% SD vs. 11%±1% SD) [21].

Differences in mat consistency were noticed between the different light conditions. The microbial mats cultivated with 730 nm, 890 nm, and the combined light were dense, whereas those grown in the dark or with 625-nm light were rather loose mats. Cyanobacteria are known to have the ability to produce extracellular polymeric substances (EPS) that aid biofilm and mat formation [44]. However, in this study, stimulation of cyanobacteria under 625-nm LED condition led to only loose mats, which might indicate that *Chloroflexus* sp. and/or *Roseiflexus* sp. enhanced under 730- and 890-nm LED conditions were directly or indirectly responsible for the formation of dense and firm microbial mats. *Chloroflexus* and *Roseiflexus* spp. have the potential to produce cellulose, which also is a known biofilm-enhancing component [45], as they possess the cellulose-related *cesA*/*celA*/*bcsA* gene set [46]. The hypothesized presence of cellulose in these dense mats is further supported by an increase of sequences representing anaerobic and putatively cellulose degrading species, e.g., SJA-28 member OTU41 (Chlorobi) and *Ruminiclostridium* sp. OTU30 (Firmicutes) in mats cultivated with 730- or 890-nm light (S3 Table). Sequences affiliated with the SJA-28 group have been reported to increase in the presence of cellulose under anaerobic methanogenic conditions [47] indicating a putative ability to degrade cellulose, which has readily been shown for *Ruminiclostridium* spp. [48].

Effects of different light conditions were thus observed visually as differences in color and consistency after an incubation period of just 20 days. Furthermore, as hypothesized, cultivation under different light conditions led to changes in the relative abundance of different community members (Fig 3) and will be discussed in the section "Effect of light on specific microbial mat members" below.

### Bacterial biodiversity in experimental mats, initial mat, and hot spring water

The relative biodiversity in microbial mats before and after 20 days of irradiation with specific light wavelengths and hot spring water were analyzed by 16S rRNA gene amplicon sequencing (Fig 3). A total of 22 samples from the microbial mats and the hot spring water were analyzed. Microbial mats incubated under five different light conditions as well as the initial mat used as inoculum were analyzed in triplicates, while surrounding hot spring water was analyzed at four different time points (0, 7, 14, 20 days). A total of 129,173±18,479 SD trimmed/processed sequences were analyzed for each of the 22 samples (S2 Table). No statistically significant differences were observed between the samples with regard to analyzed sequences, neither among light conditions nor replications (temperatures) (*p*s >0.14), except the differences of dark conditions with 625 nm and combined-light conditions (*p*s ~0.07 and ~0.03, respectively). The numbers of sequences were 143,096±9,024 under dark conditions, 113,107±16,739 under 625 nm conditions, and 126,066±5,680 under combined-light conditions. However, rarefaction curves, which almost plateau with >10,000 sequences (S9–S12 Figs), showed that the numbers of sequences and coverage were sufficient for all samples.

The diversity of the communities was assessed by the Shannon Diversity Index, Chao1, OTU richness, equitability based on 97% nucleotide sequence identity, and relative abundance of community members (Table 3). On average, 380±75 OTUs were detected in each of the different samples, and the expected OTU richness (Chao1) was well covered with 90±6%. The Chao1 richness of the number of OTUs detected in the hot spring water was significantly higher than that than in the mat samples (577±95 vs. 391±30 respectively) (*p* <0.05). Despite the higher number of obtained OTUs and greater Chao1 richness, the water samples displayed lower diversity due to weaker equitability (2.9±0.4 vs. 5.1±0.3 in Shannon Diversity Index and 0.32±0.05 vs. 0.60±0.03 in equitability for hot spring samples and the mat samples, respectively) (Table 3).

**Table 3:** Biodiversity in the different samples including experimental mats, initial mat, and hot spring water.

Sample	Shannon	Chao1	OTUs	Coverage^†^	Equitability
**dark 1**	5.07	394	351	89%	0.60
**dark 2**	5.33	412	373	90%	0.62
**dark 3**	5.22	424	371	87%	0.61
**625 nm 1**	5.19	409	348	85%	0.61
**625 nm 2**	5.32	347	326	94%	0.64
**625 nm 3**	5.44	411	381	93%	0.63
**730 nm 1**	4.78	357	332	93%	0.57
**730 nm 2**	5.19	394	355	90%	0.61
**730 nm 3**	5.36	444	396	89%	0.62
**890 nm 1**	5.13	381	338	89%	0.61
**890 nm 2**	5.12	421	370	88%	0.60
**890 nm 3**	5.40	401	378	94%	0.63
**3 LEDs 1**	5.34	375	358	95%	0.63
**3 LEDs 2**	5.18	386	357	93%	0.61
**3 LEDs 3**	5.12	416	369	89%	0.60
**IM 1**	4.71	346	326	94%	0.56
**IM 2**	4.71	374	352	94%	0.56
**IM 3**	4.64	337	324	96%	0.56
**HSW 0**	2.80	694	685	99%	0.30
**HSW 1**	2.67	613	447	73%	0.30
**HSW 2**	2.57	506	429	85%	0.29
**HSW 3**	3.36	494	385	78%	0.39
**average**	4.71	424	380	90%	0.55
**SD**	0.94	86	75	6%	0.12

^†^Coverage: the proportion of observed OTUs against Chao1 estimation

As all mat communities clearly differed from the surrounding hot spring water community, notable differences in biodiversity were observed before and after experimental cultivation (Fig 3). Shannon Diversity Index and equitability increased significantly under experimental conditions (*p*s <0.05), and changes in community composition and relative abundance of community members were observed (Tables 1 and 3). Of the 16 abundant members (≥1% relative abundance) in the initial mat samples, eight showed a decrease in relative abundance after irradiation with the combined light, whereas only three members increased in relative abundance after experimental cultivation (S4 Table). In contrast, the abundance of phototrophic aerobic and microaerobic bacteria (*Thermosynechococcus* sp. OTU7, *Chloracidobacterium* sp. OTU26, *Elioraea* sp. OTU34) decreased relative to the initial mat community (6.3%, 3.9%, and 1.7% decreased to 0.3%, 0.0%, and 0.2%, respectively). Although *Elioraea tepidiphila* was described as chemoheterotrophic [49], *Elioraea* sp. can be assumed to be photosynthetic and will be discussed in more detail in the section "Effect of light wavelength on phototrophic bacteria" below.

The observed differences in anaerobic and aerobic bacteria after experimental cultivation indicate a reduced oxygen concentration in these mats under experimental conditions, even under oxygenic photosynthesis supported by light conditions (S4 Table). This might be explained by the homogenization of the initial mat and/or the relatively low light conditions used in the experiment. Low light conditions could result in decreased cyanobacterial photosynthesis activity. Furthermore, homogenization could have led to increased oxygen consumption from abundant biomass degradation. The intensity of the experimental light was ~20% of natural sunlight intensity on a clear day, and thus more representative of conditions on a cloudy day [50]. This could explain the lower photosynthesis activity and less oxygen production in the mats in comparison to the initial mat, which was located on a horizontal, south-facing wall with abundant sun exposure and available nutrients and air/oxygen from the falling hot spring water. Additionally, the continuous irradiation and limited wavelengths represent artificial conditions not observed in natural habitats and may also be responsible for the observed decrease in phototrophic bacteria, which might rely on varying conditions of light and/or oxygen as has been indicated from diel metatranscriptome analyses for phototrophic hot spring mat community members [14,51,52]. Oxygen concentrations have been measured in alkaline hot spring microbial mats before, which clearly show oxygen supersaturation during day (light condition) and anoxic conditions during night (dark conditions) [53], leading us to hypothesize relatively anoxic conditions in the experimental mats of this study.

### Variability and temperature effects in experimental mats

In this study, changes in microbial community composition and diversity were observed in microbial mats incubated in-situ under controlled light conditions. We chose an approach with three replications to minimize the influence of natural variations in these mats, and the results will be discussed in the following sections. Due to the given conditions in natural hot spring environments it was not possible to keep temperature conditions stable among the three replications. A naturally occurring temperature gradient in the hot spring channel and the sequential set-up of the experimental devices led to temperature differences within the incubation location (Fig 2). In order to not add a second independent variable between the different treatments, we chose to allow different temperatures between replications, which simultaneously tested for the influence of the variable temperature. Significantly lower OTU richness (*p* ~0.02) and Chao1 (*p* ~0.07) were observed in device 1 (55°C) compared to device 3 (51°C) (345±10 vs. 379±11 for OTU richness, and 383±20 vs. 419±16 for Chao1, respectively; Table 3); indicating a lower microbial diversity at higher temperatures, as has previously indicated also by terminal restriction fragment length polymorphism and clone library analysis in Nakabusa hot spring [17]. However, observed differences between devices 1 and 2, as well as between devices 2 and 3 were not significant (for none of the parameters tested) (Table 3). The results suggest that the gradient of temperatures in our experiment affected the community diversity gradually along with the gradient.

Additionally, relative abundance of individual OTUs varied between the replications as seen in Fig 3 and S2 Dataset. The coefficient of variation (CV) for the most abundant members compared in Fig 3 ranged from 0.01 to 1.52 under different conditions (0.15–0.82 average) (Table 2). Part of the variation can be attributed to natural heterogeneity, whereas other parts are expected to represent specific temperature adaptations of the corresponding community member. High variation under all experimental conditions indicating a strong effect of temperature (CV >0.5) was observed, e.g., for Hydrogenedentes OTU4 and OTU21, *Fervidobacterium* sp. OTU12, "*Ca*. Chloranaerofilum sp." OTU27, and *Ruminiclostridium* sp. OTU30 (Fig 3 and S3 Table). In particular, Hydrogenedentes OTU4 showed a clear trend to higher relative abundance in device 3 (51°C) indicating a preference for lower temperatures, which correlated with its high sequence similarity (99% nt identity) to an uncultured bacterium detected from a 45–53°C microbial mat in Hillside Springs [54]. In contrast, Hydrogenedentes OTU21 (89% nt identity with OTU4), which showed an opposite trend towards higher relative abundance in device 1 (55°C), which might indicate a different optimum temperature. OTU12 also showed the trend towards higher relative abundance in device 1, indicating a preference for higher temperatures, which correlates with an optimal growth temperature of 65°C for its closest isolated relative, *Ferividobacterium riparium* (98% nt identity) [55]. Interestingly, "*Ca*. Chloranaerofilum sp." OTU27 and *Ruminiclostridium* sp. OTU30 strongly increased in only one sample, respectively (S3 Table), indicating a response to the specific combination of light and temperature (as discussed in the section "Effect of light wavelength on phototrophic bacteria" for OTU27). Other members showed high variation only under some but not all conditions. In particular, variations for phototrophic members differed between the conditions. *Thermosynechococcus* sp. OTU7, for example, showed large variations under dark conditions (0.67) but small variations under activating 625 nm and combined-light conditions (0.23 and 0.22, respectively). A similar trend was also observed for *Chloroflexus* sp. OTU10, for which smaller variations were observed under 730 nm and combined-light conditions than under dark conditions (0.29 and 0.14 vs. 0.41). For these cases, a lower variation in relative abundance under favorable light conditions could indicate a more active competitiveness, whereas relative abundance under unfavorable dark conditions was determined to be competitively passive, and more strongly affected by the other, more active members under these conditions. In contrast, *Roseiflexus* sp. OTU2 did not follow this trend, and showed a relatively small variation under dark conditions (0.06), which may be attributed to the chemoheterotrophic growth of *Roseiflexus* sp. [21]. Variations seen in the results of triplicates in the different light treatments indicate temperature effects due to a temperature gradient between the devices. Overall, temperature reduced the species richness, while the effects seen for specific members can be interpreted as direct or indirect effects.

### Microbial mat community grown under dark conditions

The Shannon diversity in mats incubated under dark conditions did not significantly differ from that in the mats incubated under light conditions (*p*s >0.24) (Table 3). Although relative bacterial abundance differed between the different treatments, most of the abundant (≥1% average sequence abundance) members in the mats grown under dark conditions were consistent with those in all conditions with LED light. One exception was OTU10 sequences representing *Chloroflexus aggregans*, which showed a considerably lower abundance under dark conditions as compared to the combined-light control (0.3±0.1% vs. 3.1±0.4%). This strong decrease under dark condition reflects this organism's preference for a phototrophic lifestyle, as well as the need for oxygen for chemotrophic growth. Chemotrophic growth in the dark has been observed in the type strain only under aerobic conditions [20], indicating that the expected anoxic conditions in the dark mats inhibited the growth of *Chloroflexus*. Interestingly, all abundant species under dark conditions were heterotrophic, and the presence of chemoautotrophic members as primary producers was not indicated, although some OTUs related to chemoautotrophs, such as *Thiobacter* sp. (OTU46, 0.8±0.1%) and *Caldimicrobium* sp. (OTU45, 0.13±0.01%), were moderately abundant (≤1% and ≥0.1%). These data suggest that the microbial mat biodiversity was mostly dictated by the biomass and nutrients introduced with the initial mat rather than primary production by autotrophs; however, although not demonstrated by culture experiments, the *Thermodesulfovibrio* sp. related to OTU9 (4±1% in dark condition) is suggested to possibly have the ability to grow autotrophically based on the existence of reductive acetyl-CoA pathway enzyme genes [56], and could have contributed to primary production in the mats under dark and anaerobic conditions.

### Effect of light wavelength on microbial mat biodiversity

**Fig 8:**
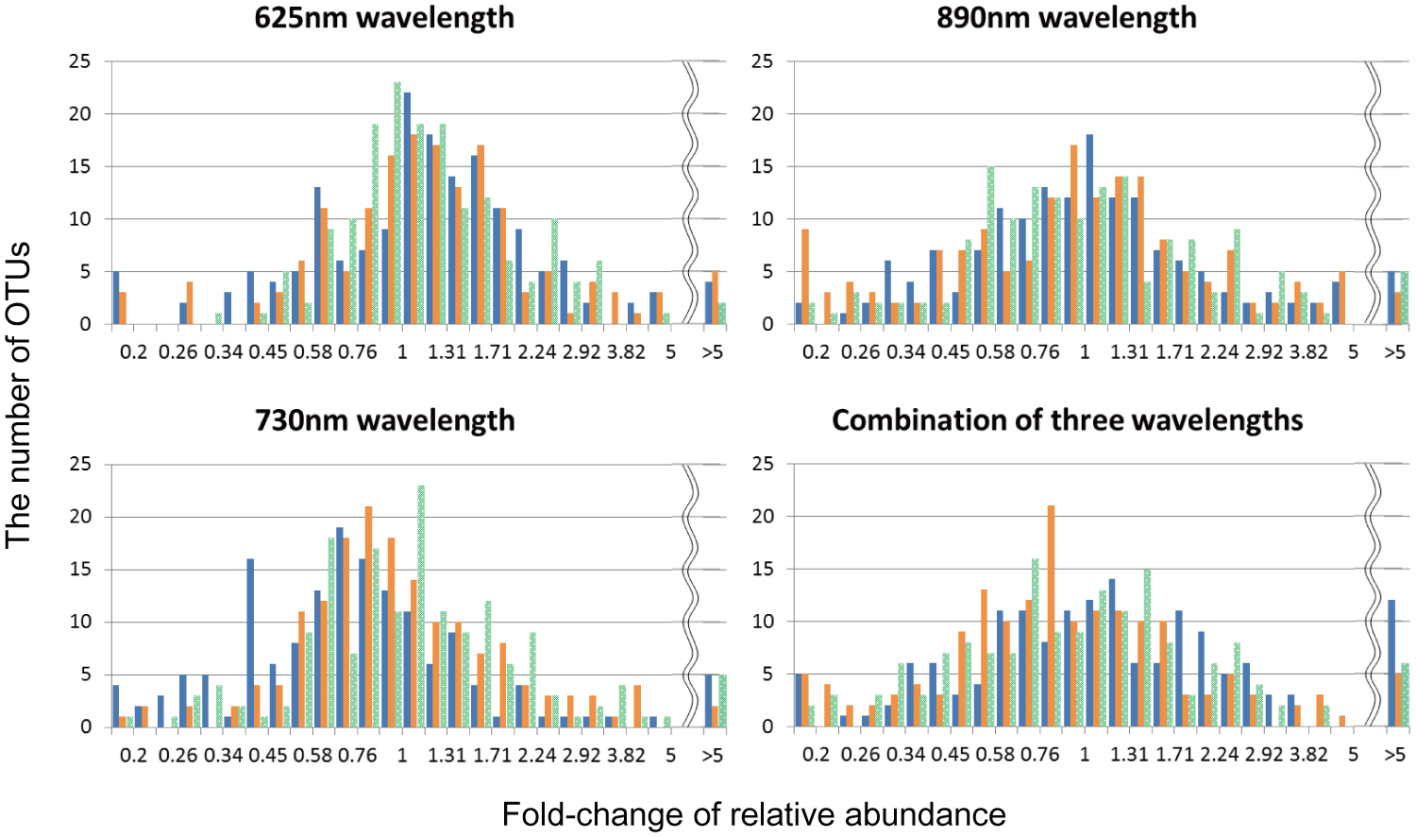
Semi-logarithmic histograms of fold changes in bacterial abundance at the indicated wavelengths. Fold changes of relative OTU abundance under the different light conditions as compared to controls grown in the dark are shown with blue, orange, and green bars for devices 1, 2, and 3, respectively. The base of the logarithm for the fold change was 5^1/12^.

Although no significant difference in species richness or Shannon diversity and equitability was detected between the different light conditions, an effect of light wavelength on microbial mat community was observed in relative abundance of different OTUs in comparison to the dark conditions, and is shown as semilogarithmic histograms in Fig 8. The average median values of the fold changes in the histograms for the 625-nm, 730-nm, 890-nm, and combined-light mats were 1.15, 0.90, 0.98, and 0.98, respectively. With an average median fold change of 1.15, the relative abundance of 62% for the OTUs in mats irradiated with 625 nm light was higher than that under dark conditions. In contrast, an average median fold change of <1.0 represents a decrease in relative abundance under the light conditions for more than 50% of the OTUs (and fewer OTUs showed a significant increase). A broadening of the histogram as seen for the 890-nm samples is indicative of more pronounced changes in abundance that are evenly distributed between the different OTUs in such a way that they average out to a median value of ~1.0. The observed changes are likely related to the most abundant photosynthetic bacteria for each wavelength (i.e., *Thermosynechococcus*, *Chloroflexus*, and *Roseiflexus* in 625-, 730-, and 890-nm samples, respectively).

The increase observed for 62% of OTUs under 625-nm LED conditions suggests that the initial community was well adapted to growing with *Thermosynechococcus*, and that the majority of the initial mat community suffers under dark conditions. Cyanobacteria produce molecular oxygen through photosynthesis and provide vitamins and organic matter, which has a profound impact on the other species within the microbiome [10,11]. As indicated by an average median value of <1.0, several bacteria decreased in abundance in mats irradiated with 730-nm light, suggesting that the increase of *Chloroflexus* under these conditions does not benefit many other community members and may result in a competitive disadvantage for other members. *Chloroflexus aggregans*, which shares 99% nucleotide identity with OTU10, consumes various types of organic matter under anaerobic light conditions, indicating that the outgrowth of this bacterium likely depletes available nutrients and manifests as the observed decrease in other heterotrophs [20].

The increase of *Roseiflexus* in 890-nm samples had equally positive and negative effects on the microbial community, as shown in a broadening of the histogram in Fig 8 and an average median value close to 1.0. Utilization of inorganic carbon sources in assumed autotrophic or mixotrophic growth by *Roseiflexus castenholzii* represented by OTU2 sequences would act as a primary producer of organic carbon and nutrients available to surrounding heterotrophs. Furthermore, this species likely participates in oxidation of sulfide and/or hydrogen based on genome information [28], which would facilitate the growth of sulfide-sensitive species and hinder the growth of species reliant on available electron donors.

### Effect of light on specific microbial mat members

In the following section we will discuss the effect of different light conditions on selected microbial community members. Due to a combination of natural heterogeneity and the introduction of a second variable (temperature) between the replications, differing variations between the replications were observed and average values are of limited reliability. We therefore focus our discussion on selected members for which a strong effect (<0.5 or >1.5 fold change from dark conditions) was observed in at least two devices (replications) of any light condition. In total, 16 OTUs met these criteria, shown in S3 Table, and will be discussed in detail here.

#### Effect of light wavelength on phototrophic bacteria

The most abundant photosynthetic bacteria observed in the present study were *Roseiflexus*, *Chloroflexus*, and *Thermosynechococcus*, which predominated in mats cultivated with 890-, 730-, and 625-nm light, respectively (Table 1). Although *Thermosynechococcus* sp. showed the most profound increase in mats irradiated with the combined light, this was not shared with *Chloroflexus* and *Roseiflexus* spp. This could indicate that the microbiome in these mats may harbor increased competition for electron donors by *Chloroflexus* and *Roseiflexus*, or that high oxygen could inhibit the growth of both phototrophic Chloroflexi, which are known to grow phototrophically only under anaerobic conditions [20,21]. Furthermore, *Chloroflexus* and *Roseiflexus* are found at almost the same depth in mats [43], suggesting that these species utilize common resources. Under combined light conditions, both filamentous anoxygenic phototrophs are activated and would therefore compete against each other for those common resources.

Similar to all cyanobacteria, *Thermosynechococcus* spp. are oxygenic chlorophototrophs that express the photosynthetic pigments chlorophyll *a* (*A*_*max*_ = 680 nm) and phycobilins (e.g., allophycocyanin in light-harvesting phycobilisomes; *A*_*max*_ = 625 nm) [18]. Although the species showed decreased relative abundance in all experimental conditions compared to the initial mat, the light conditions increased the relative abundance compared to the dark conditions. As expected given the in vivo absorption maxima of cyanobacteria, 625-nm light and the combination of all three wavelengths had the largest impact on relative abundance with a 16- and 22-fold increase, respectively. This effect was also observed visually based on the presence of a 1-mm-thick, dark green layer on top of the mats and on either side of the glass cover. Moreover, the increased sequence abundance observed at 730 nm and 890 nm could result from partial absorbance at these wavelengths, but did not manifest as visual color change on the mats.

The BChl *c* and chlorosome-containing filamentous anoxygenic phototroph *Chloroflexus aggregans* specifically increased under 730-nm and combined-light conditions, which is well in accordance with the *A*_*max*_ of BChl *c* of 740 nm in this organism [20]. As abundance of BChl *a* is clearly lower than that of BChl *c* in this organism [20], the light absorbed by BChl *a* (890 nm) had no considerable effect on the relative abundance of *C*. *aggregans*-related 16S rRNA gene sequences.

Sequences representing *Roseiflexus castenholzii*, a chlorosome-lacking filamentous anoxygenic phototroph that expresses BChl *a* as its main photopigment, increased in abundance at 890 nm and with the combined light (17±4% and 15±3% vs. 11±1% relative abundance in dark conditions), in accordance with the *A*_*max*_ of BChl *a* in this organism at 880 nm [21]. However, high abundance of *Roseiflexus* sequences was not restricted to these conditions; rather these sequences were the predominant sequences in all mats in the present study, as well as in those collected from hot springs in YNP in a previous study [40], reflecting the ability of *Roseiflexus* to grow both photo-and chemotrophically.

In addition to these three abundant photosynthetic bacteria, sequences of four less abundant (<1%) photosynthetic bacteria, namely *Elioraea* sp. (OTU34), "*Ca*. Chloranaerofilum sp." (OTU27), "*Ca*. Roseilinea sp." (OTU120), and *Chloracidobacterium* sp. (OTU26), also increased in abundance under specific experimental light conditions (S3 Table). For example, the abundance of *Elioraea* sp. increased in all conditions with LED light, and was most pronounced in the mats at 890 nm and with the combined light (all fold changes >1.5 in triplicates). Although *Elioraea tepidiphila* has been described as chemoheterotrophic [49], *Elioraea* sp. can be assumed to be photosynthetic based on the presence of all genes necessary for BChl *a* production and anoxygenic photosynthesis in the *E*. *tepidiphila* type strain genome and a related metagenome bin observed in YNP hot spring microbial mats [40]. Moreover, the *Elioraea* sp. isolate "*Ca*. E. thermophila" obtained from microbial mats in Mushroom Spring, which shares 99% 16S rRNA nucleotide identity with OTU34, has been confirmed to produce BChl *a* and grow phototrophically [57]. Based on the close relationship and the observed increase under the light conditions in this experiment, a photoheterotrophic lifestyle for *Elioraea* sp. OTU34 is assumed. OTU27 sequences related to phototrophic "*Ca*. Chloranaerofilum corporosum" (Chloroflexi) increased in the presence of light only with device 3, which had the lowest ambient temperature (51°C) (S3 Table). This may indicate a preference for lower temperatures for this organism (S4 Fig). "*Ca*. Chloranaerofilum corporosum" (OTU27, 98% nt identity) reportedly expresses BChls *a* and *c* based on metagenomic and autofluorescence studies, and has been observed to grow phototrophically under anaerobic conditions in the laboratory [57]. Thus, our observations in the present study further support the growth of "*Ca*. Chloranaerofilum sp." in Nakabusa hot spring mats. Another sequence representing a putatively phototrophic member of the community, OTU120, is related to "*Ca*. Roseilinea gracile" (96% nt identity), a BChl *a*-expressing uncultured phototroph first identified in a YNP hot spring mat [57]. OTU120 showed a ~2-fold increase in mats cultivated with 890-nm light, supporting a phototrophic life style for this organism in these mats. Interestingly, this increase was observed primarily in devices 1 and 2, which had slightly higher temperatures than device 3 (S4 Fig), possibly indicating a preference for higher temperatures. This filamentous anoxygenic phototrophic bacterium displays a need for oxygen and is affiliated with the class *Anaerolineae* within the phylum Chloroflexi [56,57].

The fourth phototrophic low abundance (<1%) member was represented by OTU26, and represents the BChl *c*- and BChl *a*-containing anoxygenic photoheterotrophic acidobacterium *Chloracidobacterium thermophilum* (97% nt identity) [58]. *Chloracidobacterium* sp. OTU26 sequences showed limited abundance in initial mat samples, but increased in all light conditions compared to the dark condition (3.0-, 2.3-, 3.0-, and 4.5-fold increases in sequence abundance with 625-nm, 730-nm, 890-nm, and combined light, respectively; S3 Table) in device 3, which exhibited the lowest ambient temperature (51°C) and corresponded to the optimum temperature of 51°C for its closest isolated relative, *C*. *thermophilum* strain B(T) (97% nt identity) [58], reflecting a phototrophic life style. Because this photoheterotrophic species expresses BChl *c*, *a* and Chl *a* with optimal absorbance at 745 nm [58], an increase in relative abundance was mainly expected with 730-nm and combined-light conditions. However, relative sequence abundance also increased in the 625- and 890-nm mats. As *C*. *thermophilum* has been shown to depend on low oxygen concentrations and cyanobacterial sequences also increased under all experimental light conditions, microaerobic conditions are hypothesized to have occurred in these mats. We confirmed the effect of various light wavelengths on phototrophic bacteria in microbial mats.

#### Light effects on chemotrophic bacteria

Light of specific wavelengths was hypothesized to show effects on chemotrophic bacteria indirectly via the activation of different phototrophic mat members. In particular, the activity of the oxygenic phototroph *Thermosynechococcus* was expected to contribute to the growth of other bacteria in mats irradiated with light of 625 nm by providing aerobic conditions and nutrients. Abundant chemotrophic bacteria (≥1% average sequence abundance) that varied in abundance in a wavelength-dependent manner included *Exilispria* sp. OTU5, *Fervidobacterium* sp. OTU12, and *Thermodesulforhabdus* sp. OTU28 (Table 1). For all three of these bacteria, an influence of oxygen is hypothesized. *Exilispira* sp. has been reported to be a strictly anaerobic, chemoheterotrophic bacterium [59], which correlates with a lower abundance under 625-nm LED conditions in which the presence of oxygen can be suspected in this experiment. Similarly, sequences related to the strictly anaerobic, chemoheterotrophic Thermotogae member *Fervidobacterium riparium* (OTU12, 99% nt identity), suggested as temperature sensitive bacterium in the section "Variability and temperature effects in experimental mats", were the least abundant (1.9±1.2%) in the 625-nm mats and most abundant at 730 nm (4.8±7.3%). This trend was clearly observed in device 1 (3.1% and 13.2%, respectively), which exhibited the highest ambient temperature of the three devices (55°C); this might reflect a preference for higher temperatures, as seen in the optimal growth of the type strain at 65°C [55]. As the oxygen produced by *Thermosynechococcus* sp. under the 625-nm condition most likely inhibited growth and given that elemental sulfur promotes *Fervidobacterium riparium* growth [55], the increased presence of elemental sulfur associated with *Chloroflexus* sp. together with the anaerobic conditions was likely responsible for its profound abundance at 730 nm. Lastly, sequences representing *Thermodesulforhabdus* sp. were most abundant in mats cultivated in the dark (3.0±0.5%) and less abundant in those irradiated at 730 nm, 890 nm, and the combined light (1.7±0.3%, 1.5±0.8%, and 1.4±0.6%, respectively). The sequences were related to *Thermodesulforhabdus* sp. M40/2 CIV-3.2 (94% nt identity) and *Thermodesulforhabdus norvegicus* (92% nt identity), which both reduce sulfate by using acetate as an electron donor [60,61]; thus, the sequences may decrease in the presence of *Chloroflexus* and *Roseiflexus*, which both also utilize acetate [14,20].

Three additional, but less abundant (<1%), sequences affiliated with chemoheterotrophic species increased in response to different light conditions: *Meiothermus* OTU33, *Thermus* OTU67, and *Caldimicrobium* OTU45 (S3 Table). Similarly, a high influence of oxygen concentrations is hypothesized for the former two of these species; but in contrast to the aforementioned species, a positive effect is postulated. *Meiothermus* and *Thermus* spp. are strict aerobic heterotrophs belonging to the Thermaceae family, and their sequence abundance increased in conjunction with *Thermosynechococcus* in 625-nm light. An interaction between *Thermosynechococcus* and *Meiothermus* has been reported previously in which *Thermosynechococcus* provides organic carbon, oxygen and reduced nitrogen to heterotrophic *Meiothermus*, and *Meiothermus* enhances the biomass production efficiency of *Thermosynechococcus* and reduces cyanobacterium-induced oxidative stress [11]. Given their high similarity to *Meiothermus*, *Thermus* spp. are hypothesized to have a similar relationship to *Thermosynechococcus*. Although cyanobacteria contribute to the growth of different heterotrophs [11,62,63], only *Meiothermus* and *Thermus* showed a manifest positive association with *Thermosynechococcus* in our experimental data. In contrast, sequences similar to those of the hot spring-derived sulfur disproportionating Thermodesulfobacteria species *Caldimicrobium thiodismutans* increased along with those of *Chloroflexus* and *Roseiflexus*, suggesting that these filamentous anoxygenic phototrophs may function cooperatively in the sulfur cycle. For instance, *Chloroflexus aggregans* and *Roseiflexus castenholzii* both oxidize sulfide via sulfide:quinone oxidoreductase activity [7,12]. Notably, none of the sequenced *Chloroflexus* strains encode dissimilatory sulfite reductase (dsr) or sulfur oxidation (sox) genes, consistent with the observation that globules of elemental sulfur are deposited outside the cells in sulfide culture medium [7,12,64]. Based on these findings, a possible sulfur-cycle mechanism present in hot spring microbial mats consisted of *Chloroflexus* and *Roseiflexus* oxidizing sulfide to elemental sulfur, which can then be disproportionated by *Caldimicrobium* [65].

In contrast, the abundance of several sequences was highest under dark conditions and decreased in response to experimental light conditions, such as the 50% decrease of *Thiobacter subterraneus* (OTU46, 100% nt identity) sequences [66] in mats irradiated with the combined light (S3 Table). *Thiobacter subterraneus* is a strictly chemoautotrophic bacterium oxidizing thiosulfate/elemental-sulfur as a sole energy source with molecular oxygen as the electron acceptor [66]. *Thiobacter* and *Caldimicrobium* spp. both utilize and compete for elemental sulfur as an electron donor. Given that *Thiobacter* utilizes oxygen whereas *Caldimicrobium* prefers anoxic conditions, it is likely that *Thiobacter* would exhibit a competitive advantage under oxygenic light conditions; however, the abundance of *Caldimicrobium* sp. sequences increased in mats irradiated with the combined light. One possible explanation for this could be a higher pH tolerance of *Caldimicrobium thiodismutans* over *Thiobacter subterraneus* indicated by their type strain descriptions [65,66], as the autotrophic growth of cyanobacteria can significantly increase the pH of hot spring microbial mats [53,67].

### Hot spring water community

The hot spring water surrounding the microbial mats is not only the chemical source for the mat community but also a possible source of bacterial seeds invading into the mats. We studied the hot spring water microbiome at different time points during the experimental incubations. The water microbiome differed significantly from the mat communities both in diversity and community composition. Although species richness was higher than that in mat samples, diversity was reduced and the community highly uneven. The water community was dominated by sequences representing a single species, the sulfur-oxidizing Aquificae member *Sulfurihydrogenibium azorense* [68] (OTU3, 99% nt identity, abundance gradually decreased from 73% to 53%), which is a common and dominant member of the chemotrophic streamer communities found at higher temperatures (67–75°C) upstream of the experimental site [17]. Additionally, sequences representing *Tepidimonas thermarum* (OTU24, 99% nt identity, abundance gradually increased from 0.01% to 10%), *Hydrogenophilus thermoluteolus* (OTU48, 99% nt identity, 2±1%), "*Ca*. Roseovibrio tepidum" (OTU29, 99% nt identity, abundance gradually increased from 0.002% to 3%), and *Thermus arciformis* (OTU67, 99% nt identity, 1.1±0.5%) were detected in the hot spring water [57,69–71] (S5 Table). The sequences obtained in the water sample rather most likely originated from white bacterial streamers observed upstream and are not adapted to the relatively lower temperatures in this experiment. However, *Tepidimonas thermarum* OTU24 and "*Ca*. Roseovibrio tepidum" OTU29 gradually increased in these conditions. Their common features can be assumed to be aerobic and adaptation to the temperature. *Tepidimonas thermarum* is strictly aerobic and its optimum growth temperature is approximately 50–55°C [69]. Furthermore, OTU29 with 99% shared nucleotide identity to the novel aerobic anoxygenic photoheterotroph "*Ca*. Roseovibrio tepidum" [57] also shared 96% nucleotide identity to aerobic *Roseomonas alkaliterrae*, which can grow at up to 55°C (optimum, 40–50°C) [72]. Their growth temperatures would be related to the temperature at the sampling location (~56°C, T1 in S4 Fig). In regards to the aerobic condition, the proportion of oxygen-producing *Thermosynechococcus* sp. and *Chloroflexus aggregans* also increased notably, from 0.001% to 2.5% and 0.1% to 2%, respectively (S5 Table). The incubation channel for this experiment has been artificially constructed and no natural microbial mat communities were present around the installed irradiation devices at the beginning of the incubation. During the incubation period and correlating with the increase of *Thermosynechococcus* and *Chloroflexus* spp. sequences detected in the hot spring water, a thin green microbial mat formed on the sediment surrounding the light-irradiating devices during that time. Thus, the *Thermosynechococcus* sp. and *Chloroflexus aggregans* sequences detected in the hot spring water likely originated from the unintended disruption of these young microbial mats during sample collection. However, one member, *Thiobacter* sp. OTU46 (0.6% in hot spring water) was not detected in the initial mat but present in the mats after 20 days of cultivation (largest in 625-nm condition, 0.8%), which might indicate invasion from the surrounding hot spring water. Further, although not detected in high abundance in the initial spring water, the low-abundance cells can be hypothesized to be the seeds for the newly grown phototrophic microbial mats observed. We therefore confirmed the possibility that the hot spring supplied not only chemical compounds but also bacteria into the mats.

## Conclusions

In this study, we examined the effect of photosynthetic bacteria on chemotrophic members in a hot spring microbial mat in situ under controlled light conditions using 16S rRNA gene sequencing. Biodiversity analysis before and after 20 days of cultivation revealed an increase in anaerobic bacteria and a decrease of relative abundance for phototrophic bacteria that could be explained by the homogenization methods and artificial light conditions. As hypothesized, mats irradiated with light at wavelengths of 625 nm, 730 nm, and 890 nm showed significant increases in the abundance of *Thermosynechococcus*, *Chloroflexus*, and *Roseflexus*, respectively. We also observed increases of other minor phototrophic bacteria with light. These results reinforce the current knowledge of phototrophs and characterize their commensal relationship with chemotrophs which shapes the mat microbiome in situ. For example, the abundance of aerobic chemoheterotrophs such as *Thermus* sp. and *Meiothermus* sp. increased, with *Thermosynechococcus* providing aerobic conditions and photosynthates. Some chemotrophs involved in the sulfur cycle such as *Caldimicrobium thiodismutans* were correlated with the increase in *Chloroflexus* and *Roseiflexus* abundance. Control of environmental conditions in natural microbial ecosystems is a powerful tool to reveal interspecies relationships because it can reproduce various environmental conditions or regulate a specific factor. To further test the hypotheses generated and fully characterize the molecular basis of these interactions, dynamical/spatial sampling of mats and environmental information under controlled environmental conditions will be performed in the future.

## Supporting information

S1 Fig The wall site and horizontal channels at Site B in Nakabusa hot spring. Nakabusa hot spring has some outlets at (A) the Wall site (36°23’20”N, 137°44’53”E) and (B) site B (36°23’33”N, 137°44’53”E).
(TIF)

S2 Fig Initial microbial mat sampling in Nakabusa hot spring. Microbial mat samples were collected from the wall site indicated with a red rectangle on May 30th, 2016. Samples were approximately 1-cm-thick and consisted of green upper and pink undermat layers.
(TIF)

S3 Fig Dispensing of the homogenized microbial mat into the light-irradiating device. Devices (a) were filled with microbial mat samples (b) and covered (c).
(TIF)

S4 Fig Temperature and pH of the experimental set up. Temperature differences between the left and right sides were negligible (<0.5 °C).
(TIF)

S5 Fig Schematic representation of the light irradiating devices. Devices consisted of five tracks for the dark, 625-nm, 730-nm, 890-nm, and combined-light conditions. Microbial mat samples were placed in the cavities, covered with a clear acrylic board, and irradiated continuously for 20 days.
(TIF)

S6 Fig Microbial mat recovery in 20 days. At the site in which we sampled the microbial mats in this experiment, the microbial mat was recovered after 20 days.
(TIF)

S7 Fig Spectral irradiance of LEDs. Confirmation of spectral irradiance for the three LEDs at 30- and 50-cm distance: (a) OSR5CA5B61P for 625 nm, (b) SX534IR-730 for 730 nm, and (c) TSHF5410 for 890 nm.
(TIF)

S8 Fig Layered microbial mat after cultivation at 730 nm in light-irradiating device 1. The microbial mats consisted of an upper, brown layer with a thickness of ~3 mm and an orange-pink underlayer with a thickness of ~2 mm.
(TIF)

S9 Fig Rarefaction curves of the Shannon Diversity Index (n = 10, Means ±1 SE). The Shannon Diversity Index was based on 97% nucleotide sequence identity of the experimental mats, the initial mat (IM), and hot spring water (HSW) samples.
(TIF)

S10 Fig Rarefaction curves of Chao1 (n = 10, Means ±1 SE). Chao1 was based on 97% nucleotide sequence identity of the experimental mats, the initial mat (IM), and hot spring water (HSW) samples.
(TIF)

S11 Fig Rarefaction curves of observed OTUs (n = 10, Means ±1 SE). Observed OTUs were based on 97% nucleotide sequence identity of the experimental mats, the initial mat (IM), and hot spring water (HSW) samples.
(TIF)

S12 Fig Rarefaction curves of equitability (n = 10, Means ±1 SE). Equitability was based on 97% nucleotide sequence identity of the experimental mats, the initial mat (IM), and hot spring water (HSW) samples.
(TIF)

S1 Table Primers used for 16S rRNA gene sequencing. (XLSX)

S2 Table Sequences processed for taxonomic classification based on 16S rRNA gene sequences. MiSeq output sequences (total number of sequences), and then we removed the Phix genome from the raw sequences (PhiX genome removed sequences). Subsequently processed sequences (trimmed/processed sequences) were clustered as OTUs.
(XLSX)

S3 Table Microbial mat members affected by light. Orange and blue highlights indicate OTU fold changes of >1.5 and <0.5, respectively. Nearest neighbors were determined by BLAST analysis of all NCBI database sequences.
(XLSX)

S4 Table Abundant members with relative abundance ≥1% in the initial mat. Orange and blue highlights indicate relative abundance increase and decrease, respectively.
(XLSX)

S5 Table Abundant members with relative abundance ≥0.5% in hot spring water. Orange highlight indicates relative abundance ≥0.5%. Nearest neighbors were determined by BLAST analysis of all NCBI database sequences.
(XLSX)

S1 Dataset The number of reads of all OTUs taxonomically assigned using the SILVA database. "INI" indicates initial mat. "HSW" indicates hot spring water samples collected on days 0, 7, 14, and 20.
(XLSX)

S2 Dataset The averages, standard deviations, and coefficient of variations between triplicates for relative abundance of all taxonomically assigned OTUs. "INI" indicates initial mat. "HSW" indicates hot spring water samples collected on days 0, 7, 14, 20.
(XLSX)
