# Supplementary figures and images for: Effect of light wavelength on hot spring microbial mat biodiversity
Source: PLoS One. 2018 Jan 30;13(1):e0191650. doi: 10.1371/journal.pone.0191650 (PMC5790269; doi:10.1371/journal.pone.0191650)

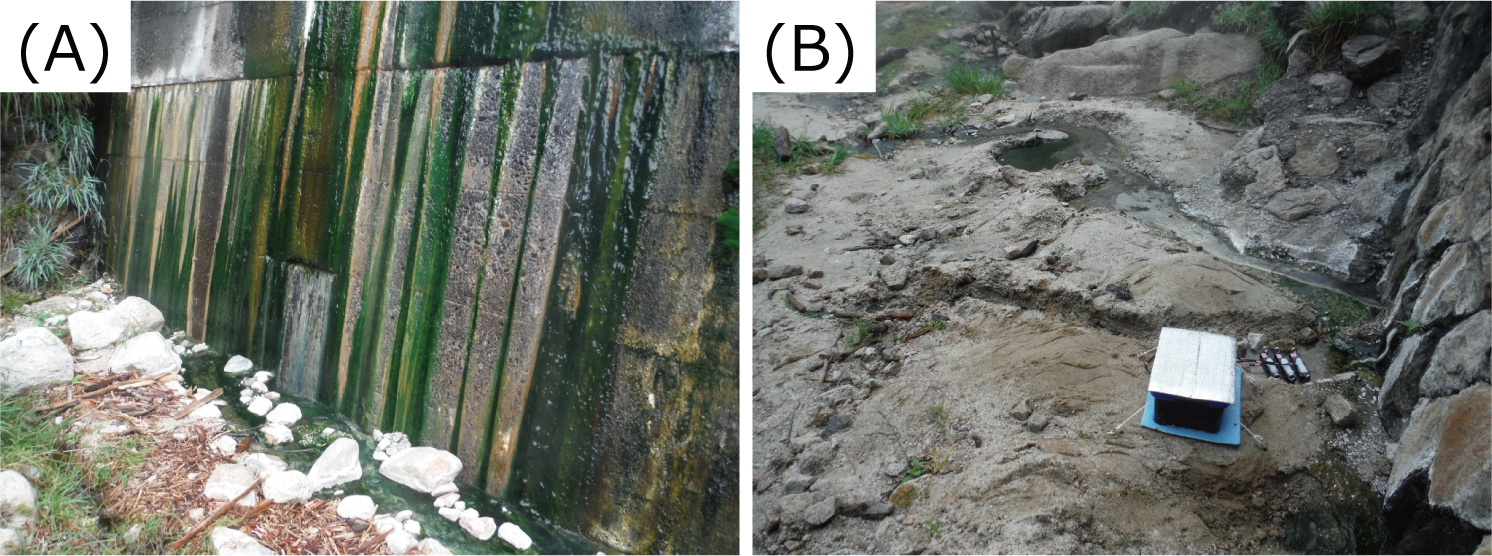

Supplement: S1 Fig — Nakabusa hot spring has some outlets at (A) the Wall site (36°23’20”N, 137°44’53”E) and (B) site B (36°23’33”N, 137°44’53”E). (TIF) [file pone.0191650.s001.tif]

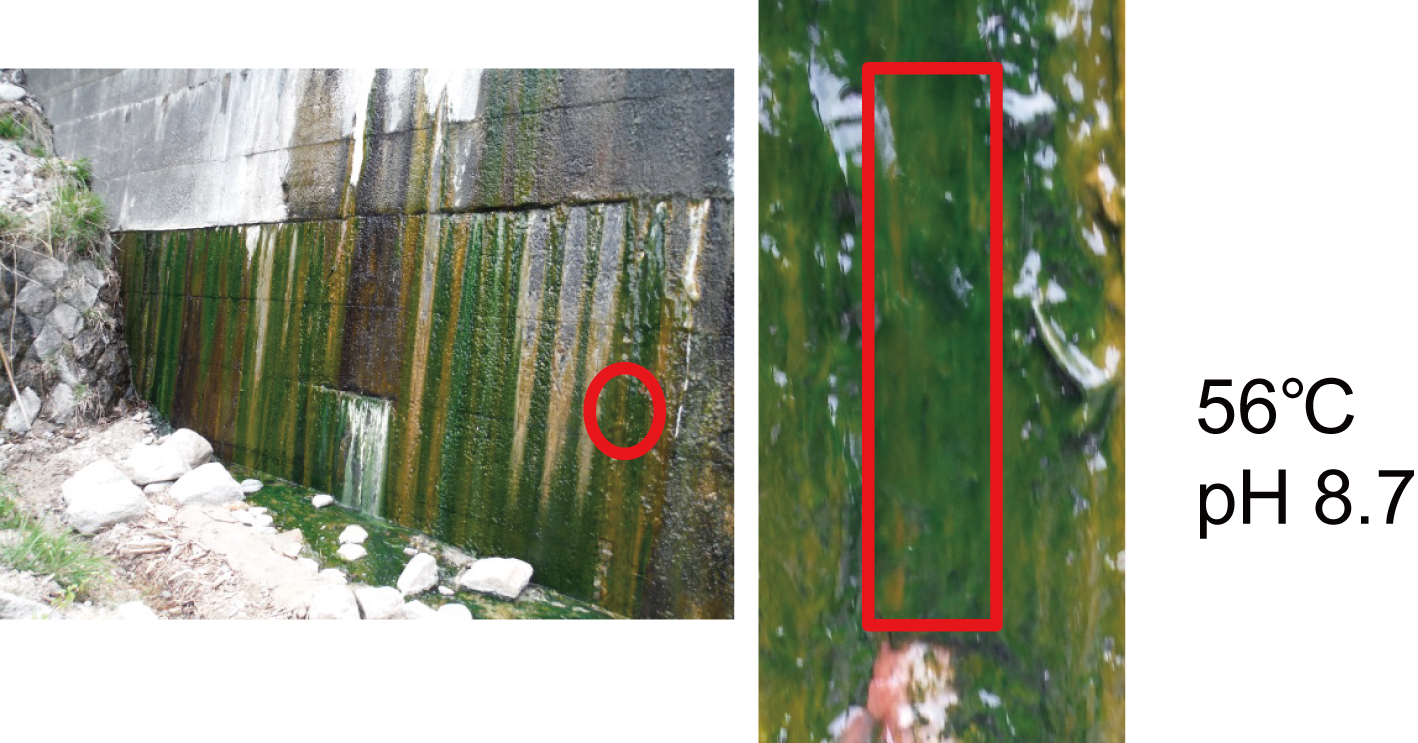

Supplement: S2 Fig — Microbial mat samples were collected from the wall site indicated with a red rectangle on May 30th, 2016. Samples were approximately 1-cm-thick and consisted of green upper and pink undermat layers. (TIF) [file pone.0191650.s002.tif]

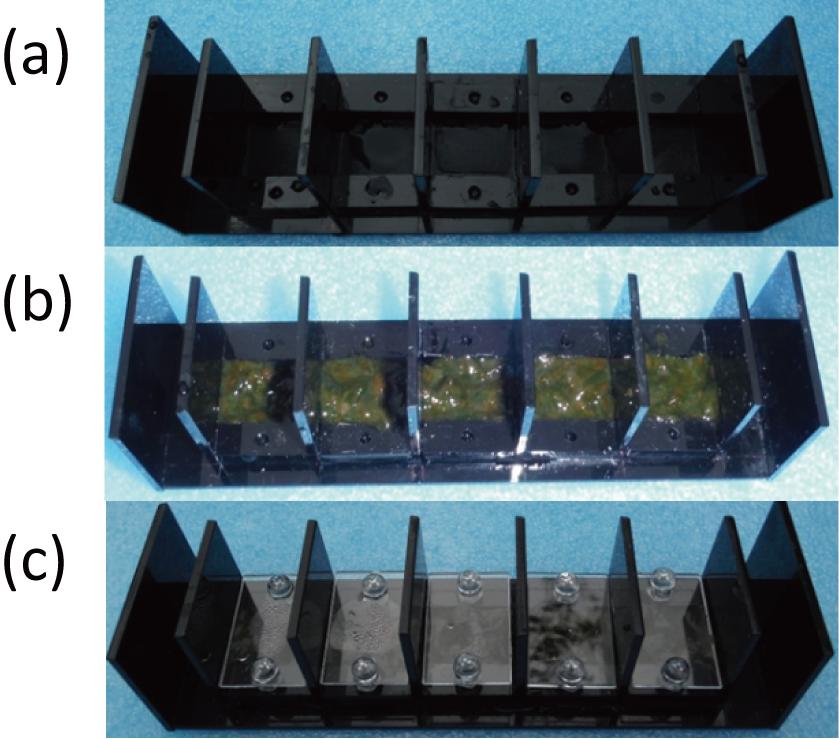

Supplement: S3 Fig — Devices (a) were filled with microbial mat samples (b) and covered (c). (TIF) [file pone.0191650.s003.tif]

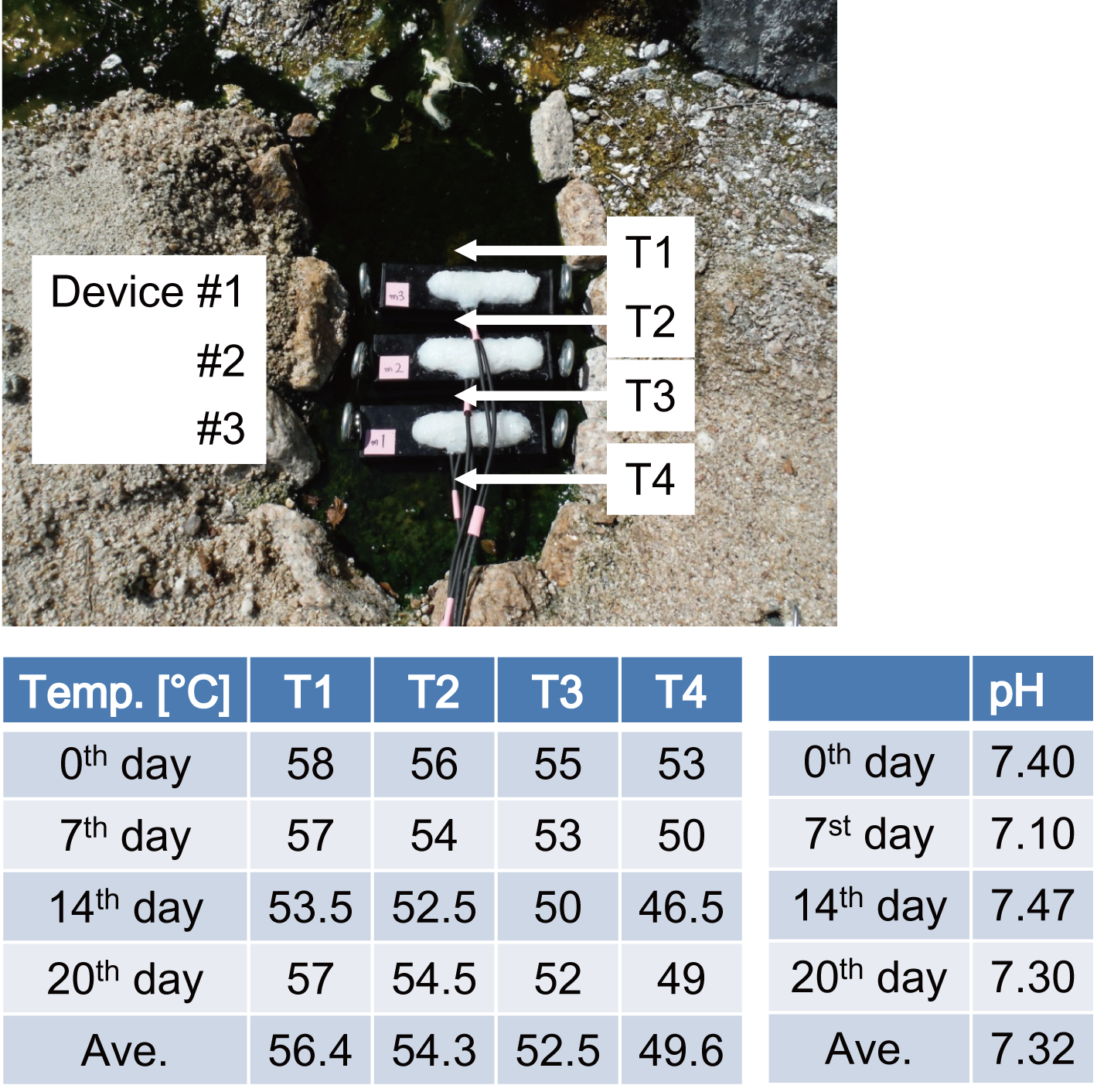

Supplement: S4 Fig — Temperature differences between the left and right sides were negligible (<0.5 °C). (TIF) [file pone.0191650.s004.tif]

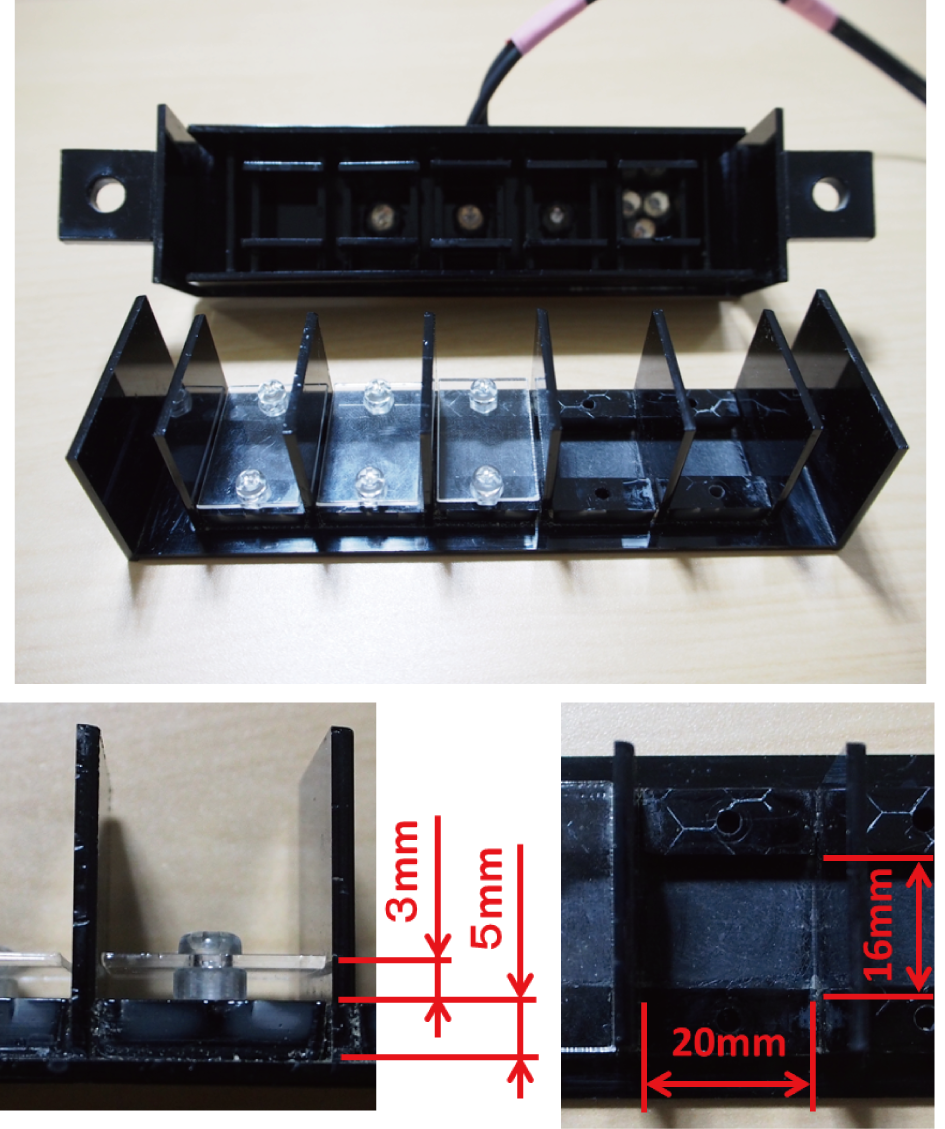

Supplement: S5 Fig — Devices consisted of five tracks for the dark, 625-nm, 730-nm, 890-nm, and combined-light conditions. Microbial mat samples were placed in the cavities, covered with a clear acrylic board, and irradiated continuously for 20 days. (TIF) [file pone.0191650.s005.tif]

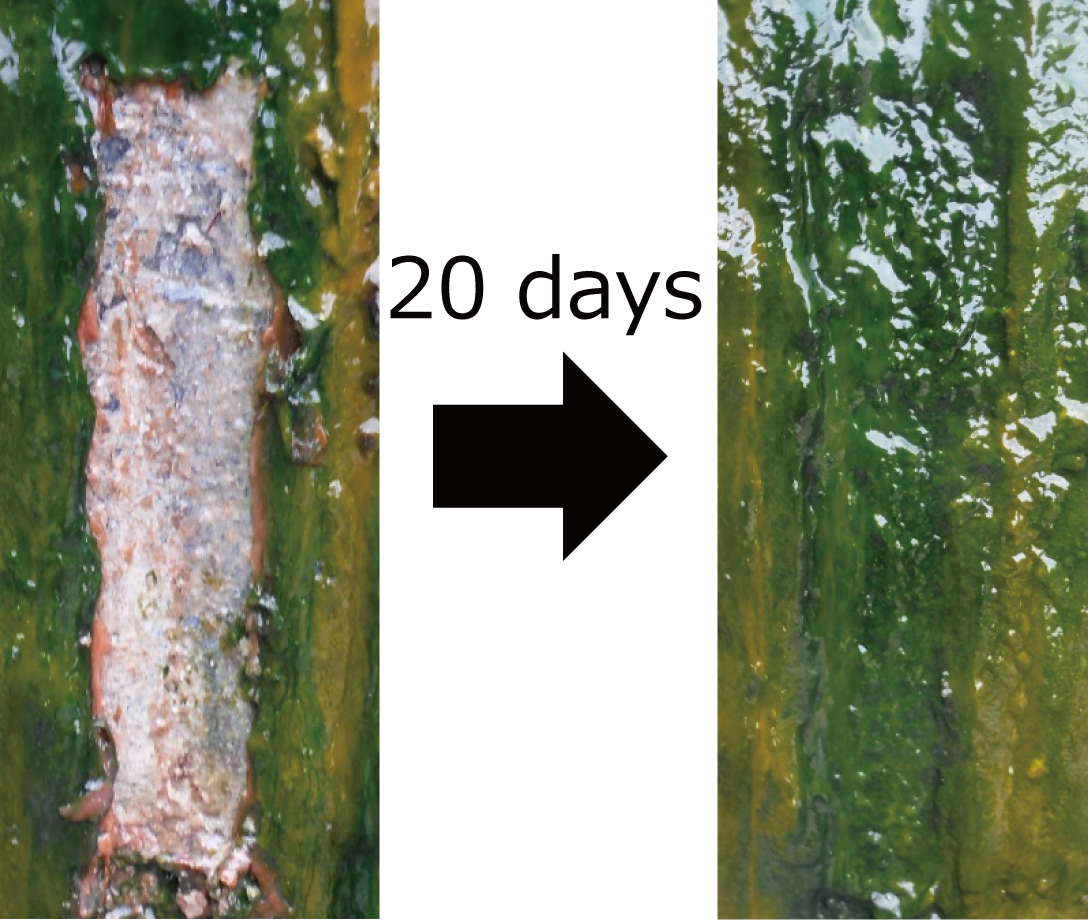

Supplement: S6 Fig — At the site in which we sampled the microbial mats in this experiment, the microbial mat was recovered after 20 days. (TIF) [file pone.0191650.s006.tif]

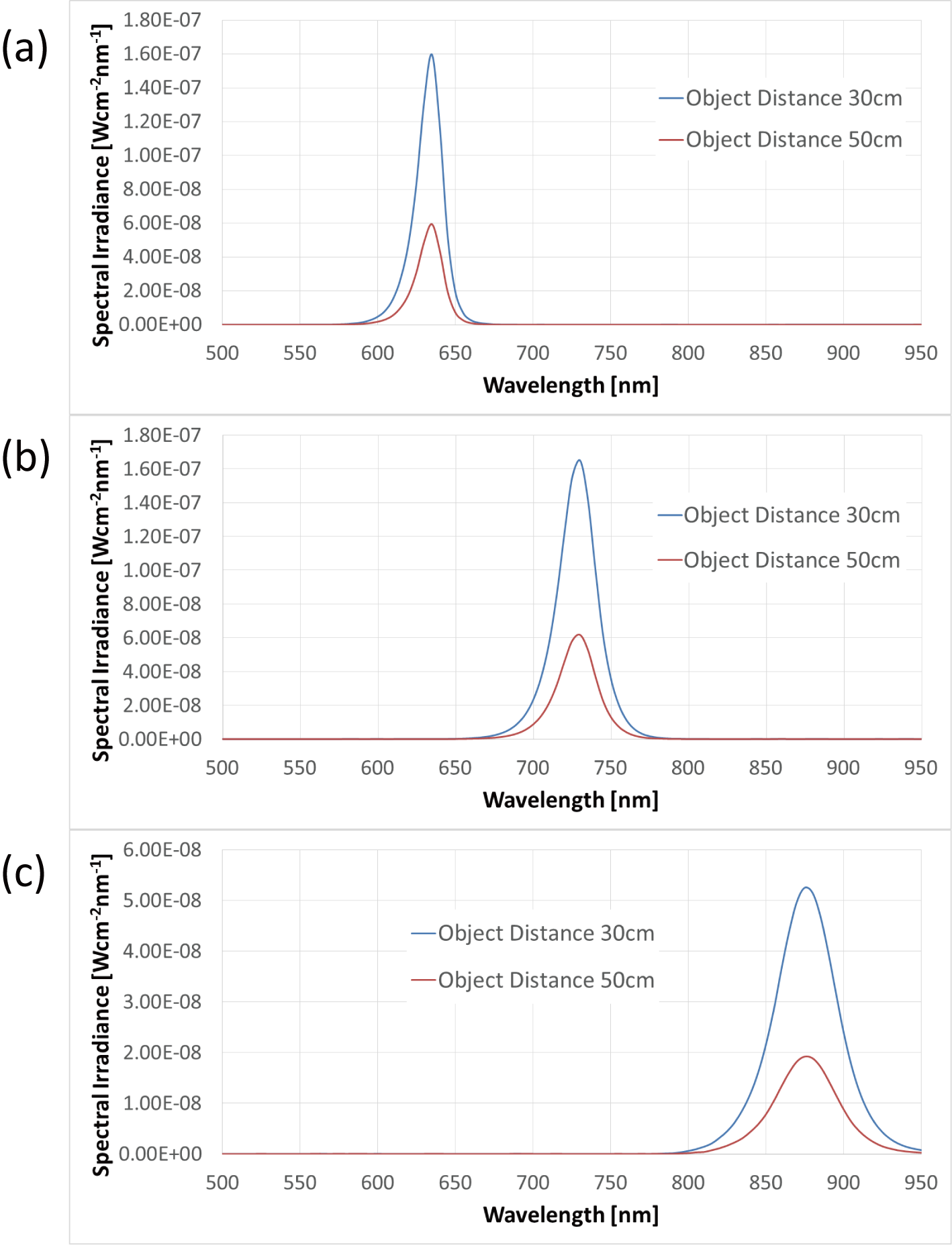

Supplement: S7 Fig — Confirmation of spectral irradiance for the three LEDs at 30- and 50-cm distance: (a) OSR5CA5B61P for 625 nm, (b) SX534IR-730 for 730 nm, and (c) TSHF5410 for 890 nm. (TIF) [file pone.0191650.s007.tif]

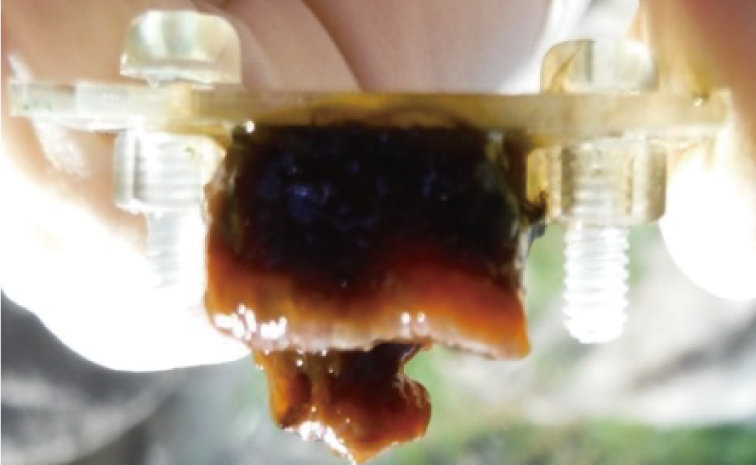

Supplement: S8 Fig — The microbial mats consisted of an upper, brown layer with a thickness of ~3 mm and an orange-pink underlayer with a thickness of ~2 mm. (TIF) [file pone.0191650.s008.tif]

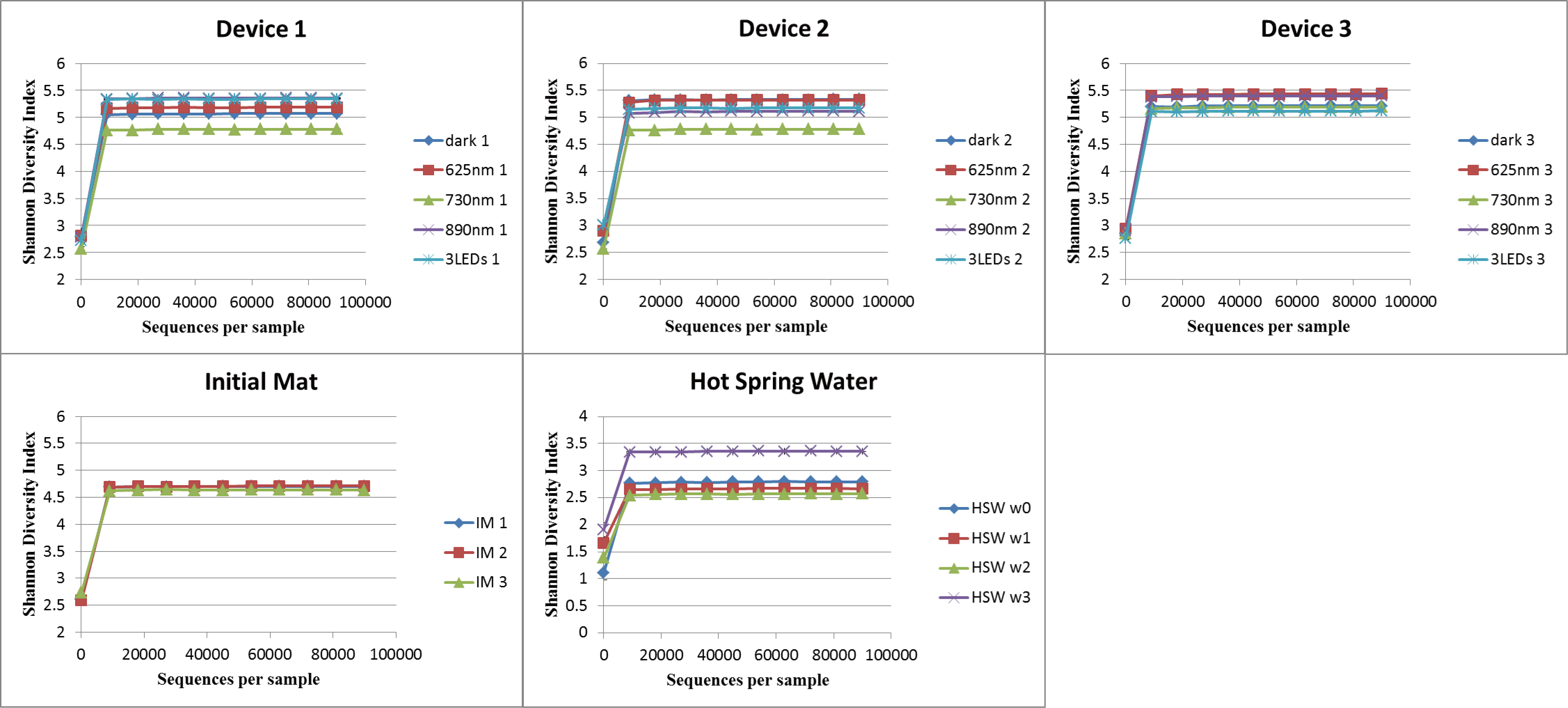

Supplement: S9 Fig — The Shannon Diversity Index was based on 97% nucleotide sequence identity of the experimental mats, the initial mat (IM), and hot spring water (HSW) samples. (TIF) [file pone.0191650.s009.tif]

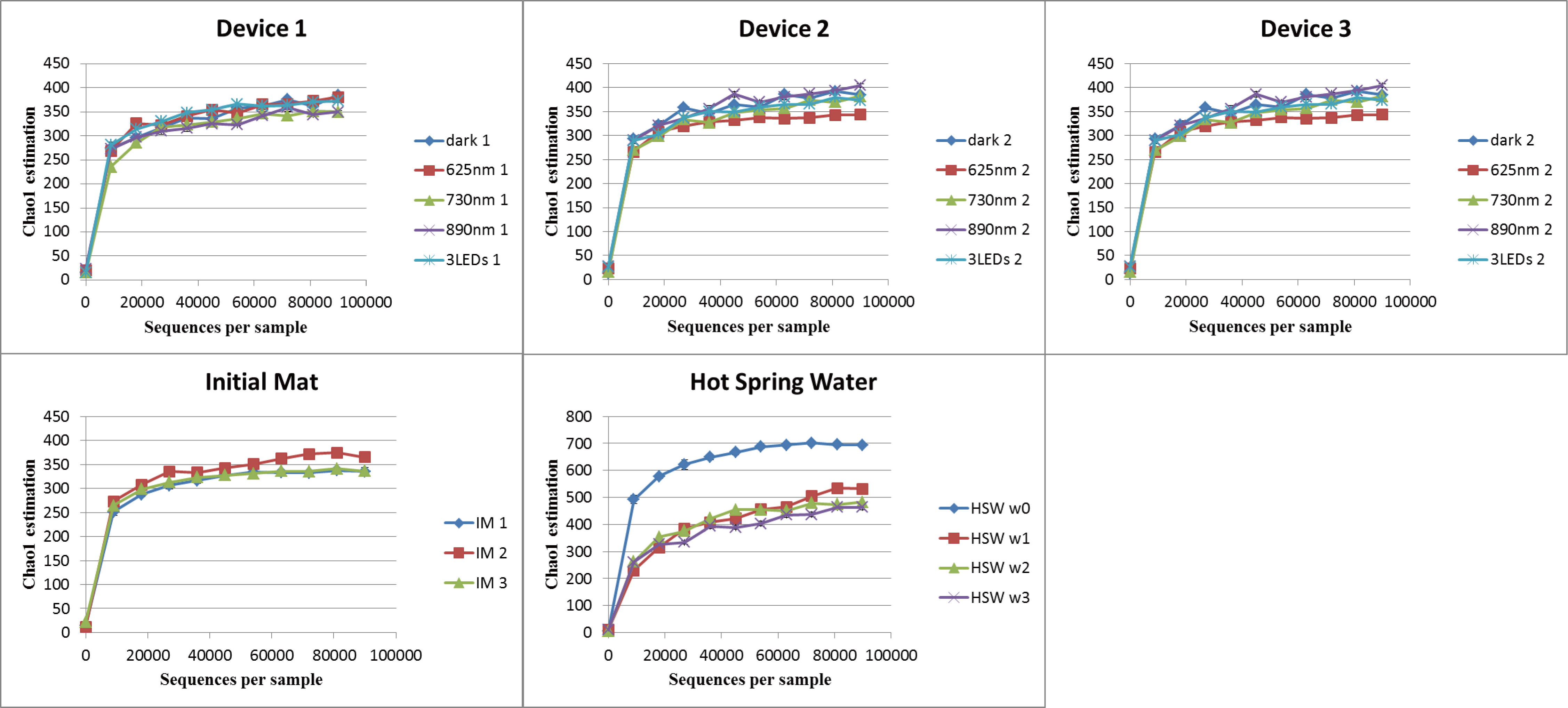

Supplement: S10 Fig — Chao1 was based on 97% nucleotide sequence identity of the experimental mats, the initial mat (IM), and hot spring water (HSW) samples. (TIF) [file pone.0191650.s010.tif]

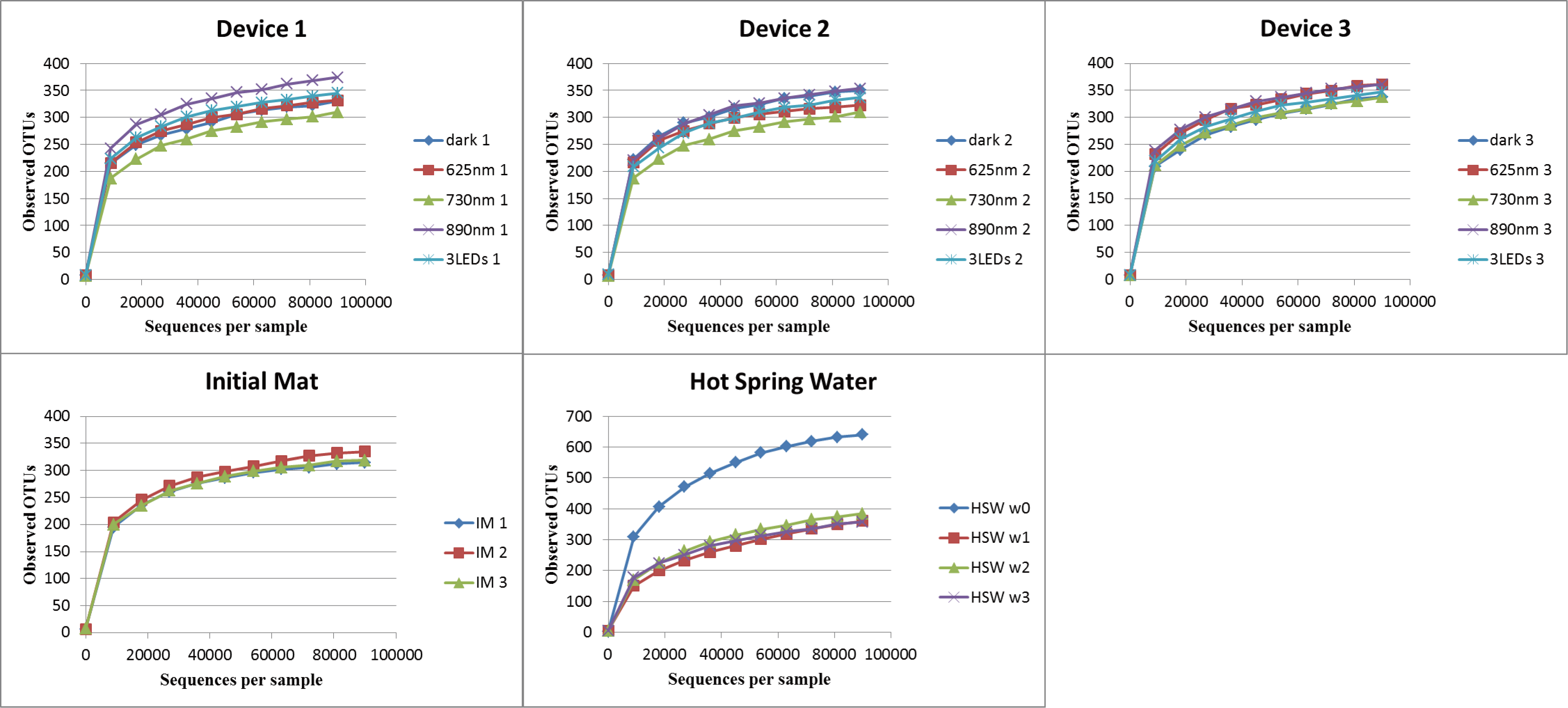

Supplement: S11 Fig — Observed OTUs were based on 97% nucleotide sequence identity of the experimental mats, the initial mat (IM), and hot spring water (HSW) samples. (TIF) [file pone.0191650.s011.tif]

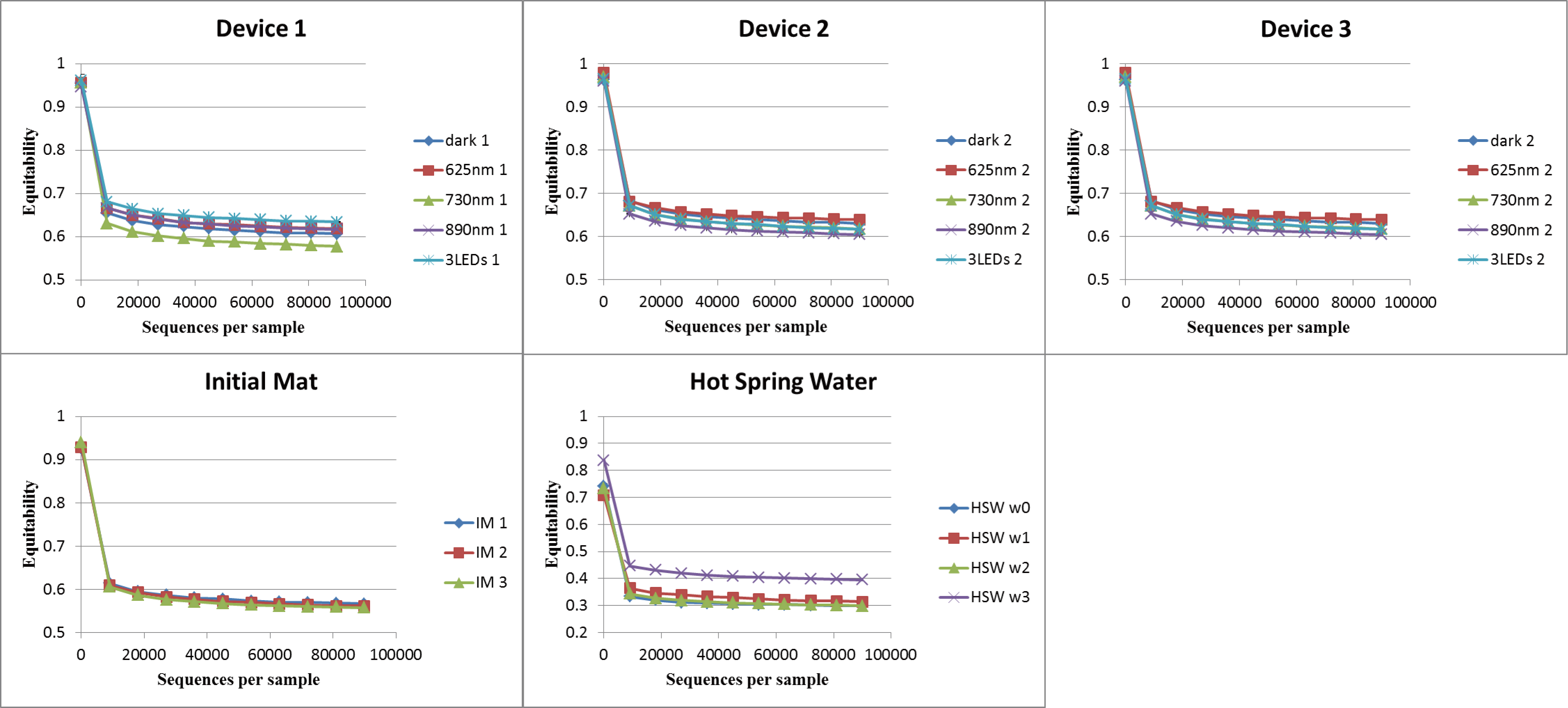

Supplement: S12 Fig — Equitability was based on 97% nucleotide sequence identity of the experimental mats, the initial mat (IM), and hot spring water (HSW) samples. (TIF) [file pone.0191650.s012.tif]
